# *In situ* and *Ex situ* Catalytic Pyrolysis of Microalgae and Integration With Pyrolytic Fractionation

**DOI:** 10.3389/fchem.2020.00786

**Published:** 2020-09-10

**Authors:** Yaser Shirazi, Sridhar Viamajala, Sasidhar Varanasi

**Affiliations:** ^1^Department of Chemical and Environmental Engineering, University of Toledo, Toledo, OH, United States; ^2^Department of Chemical Engineering, Manhattan College, New York City, NY, United States

**Keywords:** *Chlorella*, pyrolysis, zeolite, bio-oil, biochar, biofuels, HZSM-5

## Abstract

Microalgae are attractive feedstocks for biofuel production and are especially suitable for thermochemical conversion due to the presence of thermally labile constituents—lipids, starch and protein. However, the thermal degradation of starch and proteins produces water as well as other O- and N-compounds that are mixed-in with energy-dense lipid pyrolysis products. To produce hydrocarbon-rich products from microalgae biomass, we assessed *in situ* and *ex situ* catalytic pyrolysis of a lipid-rich *Chlorella* sp. in the presence of the HZSM-5 zeolite catalyst over a temperature range of 450–550°C. Results show that product yields and compositions were similar under both *in situ* and *ex situ* conditions with benzene, toluene and xylene produced as the primary aromatic products. Yields of aromatics increased with increasing temperature and the highest aromatic yield (36.4% g aromatics/g ash-free microalgae) and selectivity (87% g aromatics/g bio-oil) was obtained at 550°C. Also, at this temperature, oxygenates and nitrogenous compounds were not detected among the liquid products during *ex situ* catalytic pyrolysis. We also assessed the feasibility of a two-step fractional pyrolysis approach integrated with vapor phase catalytic upgrading. In these experiments, the biomass was first pyrolyzed at 320°C to degrade and volatilize starch, protein and free fatty acids. Then, the residual biomass was pyrolyzed again at 450°C to recover products from triglyceride decomposition. The volatiles from each fraction were passed through an *ex situ* catalyst bed. Results showed that net product yields from the 2-step process were similar to the single step *ex situ* catalytic pyrolysis at 450°C indicating that tailored vapor phase upgrading can be applied to allow separate recovery of products from the chemically distinct biomass components—(1) lower calorific value starch and proteins and (2) energy-dense lipids.

## Introduction

Microalgae are primarily comprised of starch, proteins and lipids and are especially attractive biomass resources for biofuel production due to presence of energy dense triglycerides and fatty acids (Georgianna and Mayfield, 2012). Traditional approaches of lipid extraction or *in situ* transesterification can produce fuel from only the biomass lipids (Yu et al., 2015; Skorupskaite et al., 2016). Thermochemical methods, however, can convert all organic components of microalgae into fuels or their precursors. In pyrolysis, biomass is thermally degraded in the absence of oxygen to produce gases, liquids (bio-oil), and solids (biochar) (Babu, 2008; Wang et al., 2013). Higher bio-oil yields are obtained when pyrolysis is performed with short vapor residence times and fast heating rates—a process termed fast pyrolysis (Huber et al., 2006; Bridgwater, 2012). In large scale systems, fast pyrolysis is implemented using fluidized bed, sprouted bed or ablative reactors (Bridgwater, 2012). In the laboratory, fast pyrolysis behavior of biomass is often simulated using Pyroprobe™ or similar instruments (Wang et al., 2014b, 2017; Mukarakate et al., 2015; Murugappan et al., 2016; Mullen et al., 2017). High heat transfer, precise control of temperature and short vapor residence time that are required for fast pyrolysis can all be achieved in the Pyroprobe™ instrument (Babu, 2008).

Since starch, proteins and lipids are all highly thermally labile, pyrolysis is a promising pathway to produce biofuels from microalgae (Maddi et al., 2011, 2017; Rizzo et al., 2013). However, bio-oil from microalgae biomass cannot be directly used as liquid fuel due to high heteroatom (O and/or N) content, water content, acidity and thermal/chemical instability (Elkasabi et al., 2016; Sebestyén et al., 2016; Chiaramonti et al., 2017). These undesirable properties of bio-oil are directly related to microalgae constituents (Gong et al., 2014). For instance, pyrolysis of the carbohydrate fraction from microalgae produces oxygenated compounds such as aldehydes, ketones, carboxylic acids, alcohol and water (Wang et al., 2013). Furthermore, protein constituents from microalgae pyrolysis are converted into N-compounds, and consequently the bio-oil may contain high nitrogen concentrations depending upon the N content of microalgae feedstock (Miao et al., 2004; Rizzo et al., 2013; Wang et al., 2013). On the other hand, pyrolysis of triglycerides produces bio-oil that is rich in hydrocarbons (Shirazi et al., 2016). Thus, if pyrolysis volatiles from protein and carbohydrate constituents could be collected separately from triglyceride pyrolysis products, it would allow the downstream processing of each fraction to be tailored to the different chemical and physical properties of these fractions (protein, carbohydrate, and lipid).

In recent work, we have observed that the biopolymer components (protein, starch and triglycerides) volatilize over narrow and distinct temperature regions (Maddi et al., 2017). This allows recovery of N-containing compounds and carbohydrate products from the biomass by first heating to ~320°C and holding temperature steady until a majority of the protein and starch are thermally degraded or stabilized *via* polymerization to biochar. The biomass remaining after thermal decomposition of protein and starch can be further pyrolyzed at higher temperature to recover bio-oil from the triglycerides with much lower N- and O-content (Maddi, 2014; Maddi et al., 2015a,b).

The bio-oil produced from thermal deconstruction of microalgae is typically upgraded through hydrotreating or catalytic cracking to drop-in fuel molecules (Furimsky, 2013; Jarvis et al., 2016). The hydrotreating process is conducted at elevated temperature (300–450°C) and pressure (up to 20 MPa) and requires H_2_. The bio-oil quality is improved during the hydrotreating due to a decrease in O and N and an increase in H/C ratio. However, due to high pressure H_2_ requirements, hydrotreating can incur high capital cost. Furthermore, catalyst deactivation and low catalyst lifetime (<200 h) is a challenge for commercialization of the biomass hydroprocessing (Mortensen et al., 2011). Alternatively, catalytic cracking can deoxygenate the bio-oil to produce hydrocarbons that are compatible with petro-fuels. The reaction is carried out at elevated temperature (400–600°C, similar to pyrolysis temperatures) and near-atmospheric pressure over solid acid catalysts (Thangalazhy-Gopakumar et al., 2012). Zeolites such as ZSM-5, SAPO-34, zeolite Y and β are widely used cracking catalysts due to their crystallinity, well-defined pore structures, large surface area, strong acidity and high thermal resistance (Huber and Corma, 2007; Jae et al., 2011; Rezaei et al., 2014).

Catalytic cracking is often combined with pyrolysis in either *in situ* or *ex situ* catalyst configurations and is commonly referred to as “catalytic pyrolysis” (Venderbosch, 2015). While the *in situ* approach provides intimate contact of catalyst with pyrolysis vapors as soon as they are produced and results in high yields of desired product (Wang et al., 2014a), a big challenge in this process is the recovery of catalyst after reaction is complete since the catalyst becomes co-mingled with char. Char combustion allows catalyst recovery and is a viable option for lignocellulose pyrolysis, but biochar from microalgae is N-rich and combustion produces significant NO_x_. Biochar from microalgae is also a valuable source of nutrients and, if recovered, is usable as a soil amendment and fertilizer (Yu et al., 2017).

While previous studies have investigated catalytic pyrolysis of microalgae (Kumar et al., 2017; Azizi et al., 2018), including *Chlorella* sp. (Dong et al., 2013; Du et al., 2013; Wang and Brown, 2013), most studies have used microalgae feedstocks that are relatively lipid-lean (lipid content <25% w/w). Further, comparative data on catalytic and non-catalytic pyrolysis under identical feedstock and reactor conditions is also not well reported. As such, the goals of this study were to (1) perform side-by-side comparisons of catalyst-free, *in situ* and *ex situ* catalytic pyrolysis of microalgae, (2) investigate the catalytic fast pyrolysis behavior of lipid-rich microalgae (lipid content ~40% w/w) and (3) investigate the use of catalysts in conjunction with our novel pyrolytic fractionation method that is tailored for conversion of lipid-rich algae. The pyrolytic fractionation approach provides the opportunity to develop biopolymer-specific upgrading strategies such that catalysts and/or operating conditions for fatty acid-rich bio-oils (from triglyceride degradation) and N- and O-rich bio-oils (from starch and protein degradation) may be separately optimized. Experiments were performed with *Chlorella sorokiniana* str. SLA-04 with HZSM-5 catalyst. Product yields and compositions from the novel pyrolytic fractionation coupled with *ex situ* catalytic upgrading were compared with the more traditional single step catalytic pyrolysis approach.

## Experimental

### Materials

*Chlorella sorokiniana* str. SLA-04, a natural isolate (Vadlamani et al., 2017), was mixotrophically cultivated in 750 L outdoor raceway ponds (Vadlamani, 2016). Stationary phase cultures were centrifuged (2500 × g), washed with deionized water and freeze-dried (Labconco Freezone 2.5 L bench-top freeze drying system, Kansas city, MO) to obtain the feedstock used in this study.

NH_4_-ZSM-5 powder with a SiO_2_/Al_2_O_3_ molar ratio of 2:3 was purchased from Zeolyst International, USA. The NH_4_-ZSM-5 was calcined in a muffle furnace for 5.5 h at 550°C to obtain HZSM-5. The texture and acid properties of the HZSM-5 are listed in Table S1 (supporting information) and a detailed description of HZSM-5 characterization is provided elsewhere (Shirazi et al., 2017).

Hexane, chloroform, methanol and sulfuric acid were purchased from Fisher Scientific (Pittsburgh, PA, USA). Analytical standards for fatty acids (oleic acid and palmitic acid), glycerides (triolein, diolein, and monolein), fatty acid methyl esters (FAMEs; mixtures of C_8_-C_22_), indole, pyrrole, lauramide, acetic acid, hexanoic acid, furfural, levoglucosan, pentanone, phenol, alkanes (C_5_, C_6_, C_7_, C_8_ and mixtures of C_7_-C_30_), olefins-(Alphagaz PIANO), aromatics-(Alphagaz PIANO), mixtures of benzene, toluene, ethylbenzene and xylene (BTEX) and naphthalene were purchased from Sigma-Aldrich (St. Louis, MO, USA).

### Pyrolysis-GC-MS Using Pyroprobe^TM^

**Figure 1:**
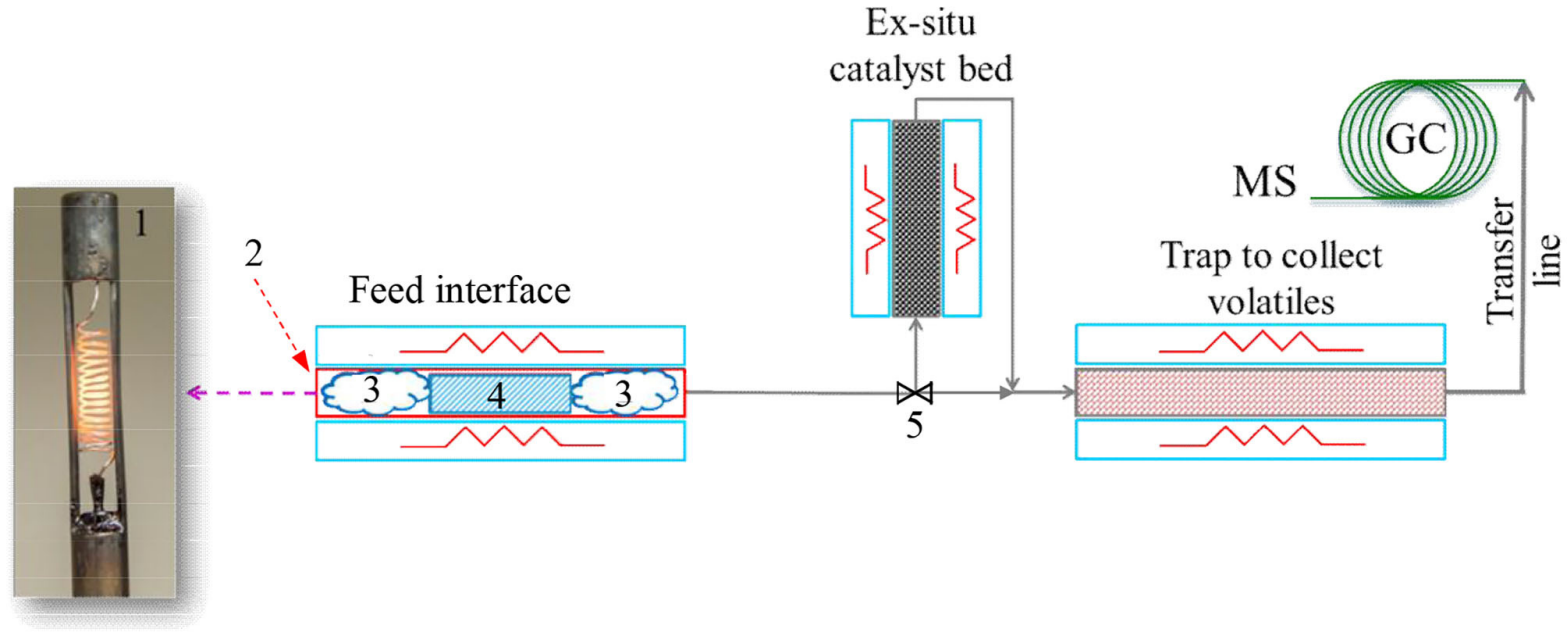
Pyrolysis micro-reactor set up with Pyroprobe-GC/MS. 1. Heating filament; 2. Quartz pyrolysis tube (placed inside the heating filament); 3. Quartz wool; 4. Biomass (mixed with catalyst for *in situ* pyrolysis); and 5. Bypass valve. Catalyst was placed in the *ex situ* catalyst bed for the *ex situ* pyrolysis experiments. Flow through the *ex situ* catalyst bed was bypassed for the *in situ* catalytic pyrolysis experiments.

In this study, pyrolysis experiments were performed on a CDS Pyroprobe™ 5,200 unit (CDS Analytical, Oxford, PA) connected to a Bruker 450 gas chromatograph (GC) equipped with a 300 series mass spectrometer (MS) (Billerica, MA) and flame ionization detector (FID). A schematic diagram of the experimental set-up is shown in Figure 1. The sample was loaded in a quartz tube that serves as a micro-pyrolysis reactor. The quartz tube was placed in a platinum heating element to rapidly heat up the sample. Furthermore, the heating element was connected to a temperature controller to maintain the micro-pyrolysis reactor temperature at the desired set point. The volatiles produced from pyrolysis of biomass were swept from the reactor (using helium as the carrier gas) and routed to a trap packed with a Tenax® adsorbent material to adsorb the volatiles from pyrolysis reaction. In pyrolysis experiments performed with *ex situ* catalyst, the volatiles from the biomass pyrolysis first passed through a catalyst bed (maintained at the same temperature as the micro-pyrolysis reactor) and thereafter the upgraded vapors were routed to the Tenax® adsorbent bed. After completion of pyrolysis, the volatiles adsorbed on the trap were desorbed (by increasing the trap temperature) and routed to the GC-MS for analysis. A heated transfer line connected the trap to the GC injector to prevent condensation of the volatiles.

### Experimental Procedure

For each pyrolysis experiment 5–7 mg of dry and accurately weighed microalgae biomass was used. For *in situ* catalytic pyrolysis, microalgae samples were mixed with HZSM-5 at a biomass/catalyst weight ratio of 1/5 and the mixture was loaded into the Pyroprobe™ quartz tube and is similar to the biomass catalyst ratios used in previous studies (Thangalazhy-Gopakumar et al., 2012; Dong et al., 2013; Du et al., 2013; Wang and Brown, 2013; Wang et al., 2014a,b). The same microalgae/catalyst weight ratio was used for *ex situ* catalytic pyrolysis, in which the catalyst was packed in the external reactor connected to the end of the Pyrolyzer section. Each experiment was performed two times and the average values are reported. In all cases, the difference in results between the replicates was <10%.

In the fractional pyrolysis experiments (with *in situ* or *ex situ* catalyst), the reaction conditions were chosen to be consistent with our previously reported optimal settings (Maddi et al., 2018). The microalgae was first pyrolyzed at 320°C for 10 min. The volatiles from this first step were passed through a catalyst bed that was also maintained at 320°C. The bio-oil from the first step was then transferred to the GC-MS for analysis. After completion of first pyrolysis step, the reactor was cooled down and the quartz tube that contained the solid residue was carefully removed from the Pyroprobe™ and weighed to determine the biochar mass. Thereafter, the solid residue was reloaded into reactor and pyrolyzed again at 450°C (in the presence of the same *ex situ* catalyst used in the first step) for 10 min and the bio-oil from the second fraction was analyzed by GC-MS. At the end, the residue from the second fraction was removed from the Pyroprobe™ and weighed. The solid residue fractions were weighed by an analytical balance (model XP6; Mettler Toledo, USA) with ± 0.01 mg accuracy. These experimental conditions were similar to our previously reported studies on pyrolytic fractionation of microalgae (Maddi et al., 2018).

The yield of pyrolysis products were calculated as:

(1) $$\begin{array}{l} {\text{Y}}_{\text{Bio}-\text{oil}}=\frac{{\text{W}}_{\text{Bio}-\text{oil}}}{{\text{W}}_{\text{AF}-\text{Biomass}}}\times 100 \end{array}$$

(2) $$\begin{array}{l} {\text{Y}}_{\text{Biochar}}=\frac{{\text{W}}_{\text{Biochar}}}{{\text{W}}_{\text{AF}-\text{Biomass}}}\times 100 \end{array}$$

where, *W*_Bio−oil_ is the weight of bio-oil produced and was calculated by adding the mass of all chemical compounds that were detected by GC-MS (see section Gas Chromatography (GC) Analysis). *W*_Biochar_ is the dry, ash-free biochar weight that measured gravimetrically and W_AF−Biomass_ is dry, ash-free biomass weight. To avoid absorption of ambient moisture, all gravimetric analyses were performed immediately after the exposure of biomass and biochar to the ambient atmosphere. Gas yields can be estimated from a simple mass balance as shown below:

$$\begin{array}{l} {\text{Y}}_{\text{Biogas}}\;=100-{\text{Y}}_{\text{Bio}-\text{oil}}-{\text{Y}}_{\text{Biochar}} \end{array}$$

### Analytical Methods

#### Feedstock Characterization

Proximate analysis was performed using dried biomass to measure volatile matter, fixed carbon and ash content of biomass. Volatile matter content was determined using a thermo-gravimetric analyzer (SDT Q600 series analyzer, TA Instruments, Schaumburg, IL) by measuring weight loss after heating biomass samples under an N_2_ atmosphere from room temperature to 575°C at a temperature ramp rate of 10°C·min^−1^ followed by holding the biomass temperature constant at 575°C for 7 min. Ash content (*f*_*ash*_) was measured by heating the oven dried biomass at 575°C for 24 h in a muffle furnace. The fixed carbon fraction was calculated by subtracting the volatile matter and ash (in percentage) from 100.

Elemental analysis was performed using a Thermo Scientific Flash 2000 Organic Elemental Analyzer equipped with an autosampler to measure C, H and N. Elemental analysis was performed on biomass and biochar samples.

Microalgae lipids (*f*_*lipid*_) were quantified as fatty acid methyl esters (FAMEs) using an *in situ* transesterification method (Vadlamani et al., 2017). Protein content (*f*_*protein*_) was calculated by multiplying elemental nitrogen content by a factor of 6.25 (Dong et al., 1993). The carbohydrate mass fraction (*f*_*carb*_) was obtained by Equation (3).

(3) $$\begin{array}{l} f_{carb}=100-f_{{lipid}^{-}}\;f_{{protein}^{-}}\;f_{ash} \end{array}$$

#### Analysis of the Pyrolysis Products

##### Gas chromatography (GC) analysis

GC-MS (Bruker, 450-GC equipped with 300-MS) analysis was performed to identify and quantify the chemical compounds in the pyrolysis products. An Agilent DB-5MS fused silica capillary column (length: 30 m, ID: 0.25 mm, and film thickness: 0.25 μm; Agilent Technologies, Santa Clara, CA) was employed. The injector temperature was held at 300°C and a split ratio of 1:100 was maintained during the analysis. Helium was used as the carrier gas and column flow was constant at 1.0 mL·min^−1^. The column temperature was first held constant at 30°C for 10 min, then heated at a temperature ramp of 10°C·min^−1^ to 300°C and finally held at this temperature for 10 min. The transfer line, ion source, and manifold were maintained at 300, 150, and 40°C, respectively. Chemical compounds corresponding to chromatogram peaks were identified using the NIST2008 mass spectral database. A minimum 70% confidence level was used as a threshold for positive identification of IDs provided by the spectral analysis software. Concentrations of positively identified chemical compounds in the bio-oil were estimated based on calibration curves developed using corresponding external analytical standards. The chemicals in bio-oil that had <70% confidence level were designated as “unidentified” and their concentration was estimated using an average slope of the calibration curves developed for the identified chemicals. It must be noted that all or nearly-all product peaks were identified (discussed later in the Results section) so that our approach of quantifying unidentified peaks is unlikely to contribute to large errors in the overall calculations of bio-oil yield. Further, the bio-oil quantification approach used here is based on methods previously reported in the literature for micro-pyrolysis experiments (Wang et al., 2014b). It is also worth noting that some protein and carbohydrate derivatives may not volatilize and reach the MS.

##### Calorific value

The higher heating values (HHV) of the biomass and biochar were calculated using empirical Equations 4 and 5, that were previously established from experimentally measured calorific values of several biomass varieties (Friedl et al., 2005). In this study, HHV of biomass and biochar were estimated from average of the ordinary least squares (OLS) and partial least squares (PLS) values (i.e., average of values estimated from Equations 4 and 5).

(4) $$\begin{array}{l} \text{HHV}\left(\text{OLS}\right)=1.87{\text{C}}^{2}-144\text{C}-2820\text{H}+63.8\text{C}\times \text{H}+129\text{N}\\ \;+20147 \end{array}$$

(5) $$\begin{array}{l} \text{HHV}\left(\text{PLS}\right)=5.22{\text{C}}^{2}-319\text{C}-1647\text{H}+38.6\text{C}\times \text{H}+133\text{N}\\ \;+21028 \end{array}$$

where, C, H and N denote the mass fractions (range of mass fraction is 0–100%) of carbon, hydrogen and nitrogen within the sample, measured from the elemental analysis. Equations 4 and 5 estimate HHV values in kJ·kg^−1^.

## Results and Discussion

### Feedstock Characterization

Table 1 shows the results from composition, proximate and ultimate analysis of *C. sorokiniana* str. SLA-04 in comparison with other *Chlorella* feedstocks reported in the literature—*Chlorella vulgaris* (lipid-lean) (Du et al., 2013; Wang and Brown, 2013) and *Chlorella pyrenoidosa* (moderate lipid) (Dong et al., 2013). Although all the listed species are *Chlorella*, their biochemical and elemental compositions vary quite significantly. This is not unexpected, since microalgae compositions are well-known to be dependent on growth conditions (Williams and Laurens, 2010). The SLA-04 strain used in this study contains nearly 40% lipid, whereas the other strains have lower lipid but higher carbohydrate and/or protein content. Ash content is also significantly different. The biochemical composition differences also reflect in the elemental analysis—SLA-04 has a higher carbon content while the lipid-lean *C. vulgaris* has the lowest C content. C/O ratio and C/N ratios of SLA-04 are also higher due to the lower relative carbohydrate (O-rich) and protein (N-rich) content.

**Table 1:** Composition, proximate and ultimate analysis of lipid-rich *Chlorella sorokiniana* str. SLA-04 and other *Chlorella* feedstocks reported in the literature—*Chlorella vulgaris* (lipid-lean) (Du et al., 2013; Wang and Brown, 2013) and *Chlorella pyrenoidosa* (moderate lipid) (Dong et al., 2013).

**Properties**	**Feedstocks**			
**Composition (wt. %)**	***C. sorokiniana***	***C. pyrenoidosa***	***C. vulgaris^**1**^***	***C. vulgaris^**2**^***
Lipid	38.3	24.3	12.3	4.7
Carbohydrate	26.7	18.9	n.a	21.0
Protein	15.6	19.8	50.3	42.5
Proximate analysis^*^ **(wt. %)**				
Volatiles	74.5	n.a	n.a	66.6
Fixed carbon	10.1	n.a.	n.a	11.6
Ash	15.4	2.0	7.6	15.6
Ultimate analysis* **(wt. %)**				
C	52.5	52.8	49.2	42.5
H	7.3	8.1	6.3	6.8
N	2.5	5.7	8.1	6.6
O	19.3	31.4	28.9	28.0
C/O	3.6	2.2	2.3	2.0
C/N	0.06	0.13	0.19	0.18

*n. a., not available*.

^1^*from Du et al. (2013)*.

^2^*from Wang and Brown (2013)*.

*dry-basis.

### Single Step Pyrolysis

In most previous studies, catalytic pyrolysis of microalgae has been performed in the presence of an *in situ* catalyst (see Andrade et al., 2018; Azizi et al., 2018; Gautam and Vinu, 2018; and references therein), where catalyst and biomass were mixed. With this approach, pristine biochar cannot be recovered and catalyst reuse is possible only if the biochar is combusted. In the case of microalgae, biochar is especially beneficial as a fertilizer and soil amendment due to the high N content (2–10% by weight; Maddi et al., 2011, 2018; Chaiwong et al., 2013; Wang and Brown, 2013; Wang et al., 2013) and thus it is desirable that pyrolysis processes be able to recover this valuable co-product. In addition, while combustion can provide heat, the microalgae biochar would produce significant NO_x_ in addition to complications in the combustion systems due to the high ash content (Jayanti et al., 2007). In contrast, pyrolysis of microalgae in the presence of an *ex situ* catalyst can produce catalyst-free biochar that can be readily recovered. As such, single step pyrolysis of microalgae in the presence of *ex situ* catalyst was performed and product yields and compositions were compared with *in situ* catalytic pyrolysis and catalyst-free pyrolysis.

#### Product Yields

**Figure 2:**
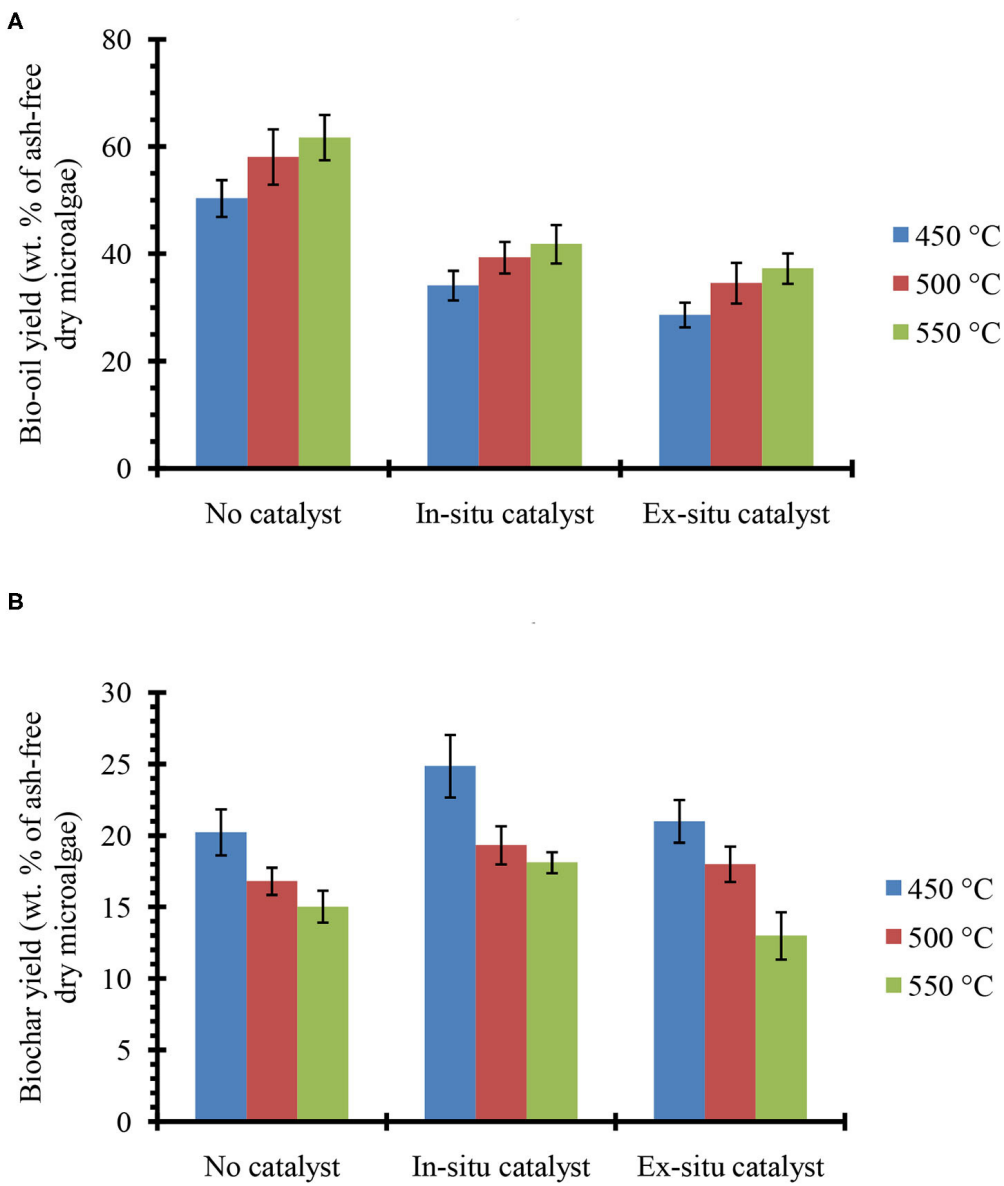
**(A)** bio-oil and **(B)** biochar yields from single step pyrolysis of microalgae in absence and presence of HZSM-5 catalyst at tested temperatures. The error bars denote the standard deviation from two experiments.

Figure 2 shows the bio-oil (Figure 2A) and biochar (Figure 2B) yields from *in situ* and *ex situ* catalytic pyrolysis of microalgae. It should be noted that in this report all the bio-oil yields are on a water-free basis since the trap on the Pyroprobe™ instrument only absorbs the organic compounds. Also the temperature values correspond to the set point temperature rather than the temperature within the sample. However, since experimental runs were long duration (10 min), it is expected that the sample temperature reached set point values. Bio-oil yield from catalyst-free pyrolysis at 550°C was as high as 62%. Previously, in non-catalytic fluidized bed experiments (fast pyrolysis) with oleaginous biomass, we also observed that bio-oil yields increased with increasing temperature and highest yields were obtained between 550 and 600°C (Urban et al., 2017). However, compared to previous studies with *Lyngbya* sp., *Cladophora* sp. (Maddi et al., 2011) and soybean flakes (Urban et al., 2017) the non-catalytic pyrolysis yields were higher likely due to the significantly higher lipid content of the *Chlorella* sp. feedstock (see Table 1) used here. In the presence of HZSM-5 catalyst, the bio-oil yields were lower, but expected due to higher cracking activities that results in production of small molecules (e.g., C_1_-C_4_) that form non-condensable gases. While these gases were not quantified here, it must be noted that catalytic pyrolysis with HZSM-5 can also produce significant amounts of olefins that could be of commercial interest (Dong et al., 2013). Previous studies in a similar micro-pyrolysis reactor set-up (*in situ* pyrolysis with HZSM-5, biomass to catalyst ratio of 1:5, and reaction temperature of 550°C) with lipid-lean *C. vulgaris* (Du et al., 2013, Table 1) resulted in bio-oil yields of approximately 22% on an ash-free dry weight basis. Our bio-oil yields under similar conditions were significantly higher at nearly 42% likely due to the much higher lipid content of the *C. sorokiniana* biomass used in this study. Catalytic pyrolysis yields also increased with increasing temperature withing the range of temperatures tested here (450–550°C) and these results are consistent with previous observations made by Brown and co-workers for catalytic pyrolysis with HZSM-5 (Wang and Brown, 2013; Wang et al., 2013, 2014a,b). It is interesting to note that the bio-oil yields from *ex situ* and *in situ* catalytic processes were relatively similar. These results suggest that an *ex situ* catalysis approach would not compromise bio-oil production while simultaneously preserving biochar and facilitating catalyst reuse.

The biochar yields decreased by increasing reaction temperature, which is similar to our previous study on pyrolysis of soybean flakes in a fluidized-bed flash pyrolysis (Urban et al., 2017). The low biochar (15–20%) and high bio-oil (50–62%) yields confirm that Pyroprobe can be a reliable instrument to simulate the fast pyrolysis conditions, where high rates of heat transfer to the biomass and short vapor residence time result in low biochar/coke formation and high bio-oil yields. Pyrolysis with *in situ* catalyst showed higher biochar compared with the experiments in the absence of catalyst, possibly due to strong acidity of the catalyst that promotes biochar/coke formation. However, in pyrolysis with *ex situ* catalyst where biomass was not in contact with the catalyst, biochar yields were similar to catalyst-free pyrolysis. After completion of the reaction, the catalyst used in the *ex situ* pyrolysis was recovered and combusted in the TGA instrument (at 600°C using ambient air) to measure the coke deposited on the catalyst. The TGA measurements confirmed that only small amount of coke (2–4 wt. % relative to biomass) were deposited on the catalyst. Low coke formation on the HZSM-5, would increase the catalyst lifetime and prevent frequent regeneration that would be required for *in situ* catalytic pyrolysis. Previous studies have shown that coke formation is the primary cause for deactivation of HZSM-5 (Cordero-Lanzac et al., 2018) and catalyst activity has been demonstrated to fully recover upon combustion of coke (Cordero-Lanzac et al., 2018; Shirazi, 2018; Shirazi et al., 2019).

#### Bio-Oil Compositions

Bio-oil compositions for the pyrolysis reactions without- and with- catalyst are shown in Table 2 [corresponding GC-MS chromatograms are in Figure S1, Supplementary Information (SI)]. In the absence of catalyst, the bio-oil was comprised of oxygenated compounds such as acetic acid, ketones, aldehydes and furans that were likely produced from the decomposition of carbohydrate constituents; N-compounds such as pyrroles, indole, pyrazoles, oleoamides and fatty nitriles produced from protein decomposition; as well as glyceride, fatty ester, aliphatic and fatty acids (mainly octadecanoic, hexadecanoic, heptanoic and octanoic acid) that are produced from decomposition and/or volatilization of lipid constituents in microalgae (Maddi et al., 2011, 2018). Moreover, by increasing the pyrolysis temperature, glyceride yields decreased, but carboxylic acid and aliphatic content increased, due to more decomposition of glyceride at higher temperatures (Shirazi et al., 2016).

**Table 2:** Bio-oil composition from pyrolysis of microalgae at tested reaction temperatures.

**Compound** 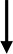	**Catalyst-free pyrolysis**			**Pyrolysis in presence of catalyst**					
				***in situ***			***ex situ***		
**T (**°**C) 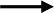 **	**450**	**500**	**550**	**450**	**500**	**550**	**450**	**500**	**550**
Benzene	0.8	1.5	1.7	2.8	4.9	5.2	3.8	6.8	7.6
Toluene	0.5	0.5	0.8	7.3	9.8	11.8	9.2	13	14.2
Ethylbenzene	–	–	–	1.6	1.8	1.9	0.2	0.4	0.5
Xylene	0.2	0.1	0.2	5.3	7	7.8	5.2	6.1	7.4
C8+ aromatics	–	–	–	4.2	4.8	4.8	1.3	0.5	0.5
Naphthalene	–	–	–	4.5	3.9	4	2.3	4	5.1
Aliphatic hydrocarbons	9.3	14.8	24.1	5.8	4.3	3.8	4.8	3.1	2
Acetic acid	2.4	1.6	1.1	–	–	–	–	–	–
Fatty acids	7.9	13.9	8.1	1.3	0.6	–	0.9	–	–
Furans	0.7	1.4	3.7	0.1	0.5	1.3	0.4	0.6	–
Other ketones/aldehydes	4.5	4.2	3.6	0.6	0.6	0.3	0.5	0.1	–
Indane	–	–	–	0.4	0.5	0.4	–	–	–
Indene	–	–	–	0.4	0.6	0.6	–	–	–
Other N-compounds	1.1	2.1	3.5	–	–	–	–	–	–
Alcohols	1.9	1.7	2.3	–	–	–	–	–	–
Fatty esters	1.5	1.3	2.9	–	–	–	–	–	–
Glycerides	15.4	10.5	4.3	–	–	–	–	–	–
Unidentified	4.2	4.5	5.4	–	–	–	–	–	–
Total liquid products	50.4	58.1	61.7	34.3	39.3	41.9	28.6	34.6	37.3
BTX^#^	1.5	2.1	2.7	15.3	21.7	24.7	18.2	25.9	29.2
Total aromatics	1.5	2.1	2.7	26.4	33.3	36.4	22.1	30.7	35.3
Aromatic carbon yield				38.3	48.3	52.8	32.1	44.5	51.2

# *Benzene, toluene and xylene*. *The values are average of two experiments and are reported as weight percentage relative to dry, ash-free biomass (i.e. % wt. product/wt. ash-free biomass)*.

In the presence of HZSM-5 (both *in situ* and *ex situ*), the bio-oil was mainly comprised of aromatics such as benzene, toluene and xylene (BTX), C_9_-C_10_ alkylaromatics and naphthalene, aliphatics (C_5_-C_18_) and small amount of oxygenated compounds such as phenol, aldehyde, furan and fatty acids. N-compounds were not observed in the bio-oil likely due to the formation of ammonia as has been previously reported (Wang and Brown, 2013). Formation of aromatics occurs due to Diels-Alder reaction and/or intramolecular radical cyclization. In the Diels-Alder reaction, dienes and alkenes react and form polysubstituted cyclohexenes which then undergo dehydrogenation to produce polysubstituted aromatics (Kubátová et al., 2011). The strong Brønsted acid sites in the HZSM-5 enable the oligomerization of light olefins (produced for dehydration, decarbonylation, decarboxylation and decomposition of microalgae volatiles) to form C_4_-C_10_ olefins which then dehydrogenate to form dienes. As described recently (Kumar et al., 2017), each of the microalgae components undergo various pathways to olefins—(1) oxygenates from carbohydrate degradation undergo deoxygenation followed by cracking, (2) proteins undergo deamination followed by cracking to aromatics and (3) lipids undergo decarboxylation and decarbonylation to long chain hydrocarbons followed by cracking to shorter olefins. Thereafter, dienes and olefins undergo cyclization and dehydrogenation to form aromatics.

The aromatics' yields were higher at higher reaction temperature. In particular, the BTX yields increased from 15 to 25% with the *in situ* catalyst and up to 29% (relative to dry, ash-free biomass) with the *ex situ* catalytic process when temperature was increased from 450 to 550°C. Interestingly, total aromatic yields were higher during *in situ* pyrolysis but BTX yields were higher when *ex situ* catalyst was used and there was no measurable evidence of oxygenated compounds under these conditions at 550°C. Figure S2 (SI) shows the selectivity of benzene, toluene and xylene from the *in situ* and *ex situ* catalytic reactions at the temperature range of 450–550°C. As observed, selectivity of benzene and toluene is higher when an *ex situ* catalyst configuration was employed. Our observations are similar to the previous report by Wang et al. (2014a) where *in situ* and *ex situ* pyrolysis of lignocellulosic biomass was compared in a micro-pyrolysis reactor like the Pyroprobe used in this study. Wang et al. (2014a) attributed the lower total aromatics, but higher BTX, yields to more rapid desorption of products in the *ex situ* configuration where the catalyst bed was smaller (due to absence of biomass) than the *in situ* reactor and possibly allowed better flow of carrier gas. In the *in situ* configuration, however, greater contact time (due to more flow resistance) between product vapors and catalyst could have allowed greater cyclization that formed more but larger aromatics. Further, in the *ex situ* catalyst configuration, HZSM-5 is expected to remain active for a longer period since the biochar and catalysts are in separate reactors. Under these reaction conditions, the catalyst acid surface and inner pores are likely more accessible and active for reaction with the volatiles that are produced from the thermal decomposition of biomass, whereas during *in situ* catalysis, the biochar formation could potentially occlude the catalyst surfaces. Our results (Table 2) showed that BTX yields were higher during ex *situ* reactions. BTX contribute greatly to the world market for commodity chemicals and have diverse uses across several industries. For instance, benzene is used as precursor for styrene, phenol, nylon and aniline production; toluene is blended into unleaded gasoline to improve the octane number; and xylene is used to produce polyethylene terephthalate (PET) and resins.

In addition to bio-oil and total aromatic yields, it is also common in the literature to report aromatic carbon yields, i.e., moles of C in the aromatic products relative to the moles of C present in the feed. For our catalytic pyrolysis experiments, the aromatic carbon yields are shown in the last row of Table 2 and were calculated using Equation (4).

(6) $$\begin{array}{l} Y_{aromatic-C}=\frac{W_{aromatics}}{W_{AF-Biomass}}\times \frac{W_{AF-Biomass}}{W_{Biomass}}\times \frac{W_{Biomass}}{W_{Biomass-C}}\\ \;\times \frac{W_{Aromatics-C}}{W_{aromatics}} \end{array}$$

Where, *W*_*aromatics*_ is the mass of aromatics produced during pyrolysis, *W*_*Biomass*_ is the mass of biomass, *W*_*Biomass*−*C*_ is the carbon mass of the feed and *W*_*Aromatics*−*C*_ is the aromatics carbon mass of the product. When written out as the ratios shown in Equation (4), the first term represents the aromatic yields from pyrolysis on an ash-free biomass basis (values given in the bottom part of Table 2). The second and third terms can be estimated from biomass ash content and carbon content values given in Table 1. The fourth term is the carbon content of the aromatic products and an approximate average value of 0.9 was used. From Table 2, our aromatic carbon yields varied between 32-53%, with higher yields at higher temperatures. Wang and Brown (Wang and Brown, 2013) reported a highest aromatic carbon yield of 23% from *in situ* pyrolysis in a micro-pyrolyzer at 700°C and a biomass to catalyst ratio of 1:20. At conditions similar to the experiments reported here (500°C and a biomass to catalyst ratio of 1:5), their aromatic carbon yields were lower at 15%. Du et al. (2013) reported aromatic carbon yields of 30% from *in situ* micro-pyrolysis experiments at 550°C using a biomass to catalyst ratio of 1:5. Under similar conditions, we observed much higher aromatic carbon yields of nearly 53% (Table 2), but it must be noted that the feed used in studies by Du et al. and Wang and Brown were lipid-lean (Table 1). Other experiments with *Chlorella* sp. and HZSM 5 have been performed in larger fixed-bed reactors with higher biomass loadings. Experiments reported by Dong et al. (2013) were performed with 5 g biomass at 600°C with a catalyst to biomass ratio of 1:20. Relatively low aromatic carbon yields were observed in this study (20% under *ex situ* catalysis), in spite of the feed containing moderate amounts of lipid (Table 1) likely due to the low heating rates (10°C/min) used which suggest slow pyrolysis. Thangalazhy-Gopakumar et al. (2012) reported aromatic carbon yields of 15–16% at 500°C from *in situ* catalytic fixed bed experiments (5 g biomass and biomass to catalyst ratio between 1:4 and 1:9) with lipid-lean biomass. By comparing our results with previously reported studies, it is apparent that high lipid content coupled with fast pyrolysis conditions are essential for high yield production of aromatics from microalgae.

#### Biochar Elemental Analysis

Biochar elemental composition from *ex situ* catalytic pyrolysis experiments is shown in Table 3. Biochar properties from catalyst-free experiments is nearly identical to the *ex situ* catalytic pyrolysis (since reaction conditions for the biomass are the same) and is not shown. Biochar from the *in situ* process was not analyzed since it is mixed with the catalyst and difficult to separate. The N content in biochar was lower at higher temperature possibly due to cracking of the C-N bonds and release of ammonia (Wang and Brown, 2013; Wang et al., 2013). The N mass balance indicates that 16–37% (16% at 550°C and 37% at 450°C) of the N content in microalgae was fixed in biochar, possibly as high molecular weight N-heterocyclic compounds (Wang et al., 2013). Moreover, there was no evidence of N-compounds in the bio-oil, indicating that a large fraction, if not all, of the remaining N formed the non-condensable ammonia as was previously reported (Wang and Brown, 2013). The biochar from *ex situ* catalytic process is catalyst-free and in addition to high N, potentially contains high amounts of other nutrients (e.g., P, K, N, S, and Ca) due to its high ash content. The C/N ratio ranges from 17-27% and is appropriate for biochar use as soil amendment or fertilizer (Verheijen et al., 2009; Trinh et al., 2013).

**Table 3:** Elemental analyses of biochar was obtained from microalgae pyrolysis in presence of the *ex situ* catalyst.

**Ultimate analysis (wt. %)**	**Temperature (**^****°****^**C)**					
	**450**		**500**		**550**	
	**Dry**	**Dry, ash-free**	**Dry**	**Dry, ash-free**	**Dry**	**Dry, ash-free**
C	32.3	66.1	31.1	69.0	27.1	72.9
H	1.2	2.4	0.9	2.1	0.5	1.3
N	2.2	4.4	1.9	4.1	1.2	3.2
C/N	17.4	17.4	19.5	19.5	26.8	26.8
H/C	0.4	0.4	0.4	0.4	0.2	0.2
HHV (MJ/kg)	16.4	24.1	16.5	24.7	16.7	24.9

“*Dry-basis” values were obtained directly from elemental analysis. “Dry, ash-free basis” values were calculated by using “dry-basis” values, and ash content. Calorific values (HHV) were calculated using Equations 4 and 5. All values are reported as mass fractions (%)*.

### Fractional Pyrolysis of Microalgae

The primary components of microalgae—starch, protein and lipids volatilize over distinct temperature ranges. Starch, protein and free fatty acids volatilize/deconstruct at temperature ranges of 160–340°C, however, microalgae triglycerides volatilize/degrade at higher temperature (see Figure S3A and previous work Maddi et al., 2017, 2018). These distinct volatilization temperatures of microalgae constituents allows collecting the products from starch/protein degradation separately from triglyceride pyrolysis products. Thereafter, each fraction can be upgraded separately *via ex situ* catalysis. To demonstrate this pyrolytic fractionation of microalgae coupled with *ex situ* upgrading, a two-step thermal treatment of *Chlorella sorokiniana* str. SLA-04 microalgae was performed in presence of *ex situ* HZSM-5 catalyst (see section Experimental Procedure). In brief, the microalgae volatiles produced from each fraction were passed through a catalyst bed that was also maintained at the same temperature as the pyrolysis reactor. A microalgae/HZSM-5 weight ratio of 1:5 was also used for these experiments. For a control experiment, the 2-step treatment was performed in the absence of external catalyst.

#### Products Yields

**Figure 3:**
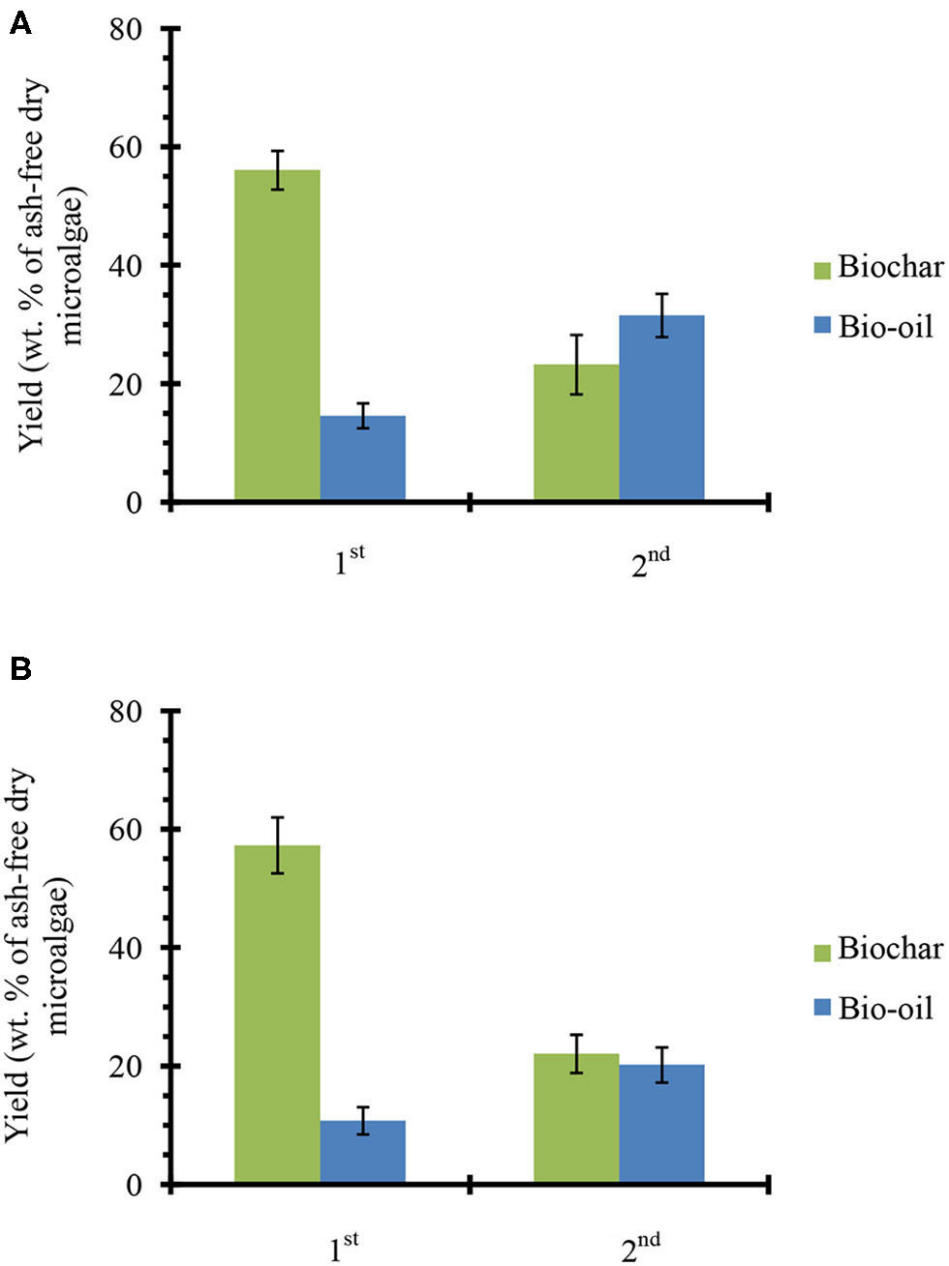
Products yields from microalgae fractional pyrolysis **(A)** catalyst-free and **(B)** with *ex situ* catalyst. The error bars denote the standard deviation.

Figure 3 shows the product yields from catalyst-free fractional pyrolysis (Figure 3A) and fractional pyrolysis with *ex situ* conversion of bio-oil vapors (Figure 3B). From catalyst-free pyrolysis, nearly 56% biochar and 15% bio-oil was produced from the first fraction (320°C). A similar residue weight fraction (~60%) was observed when the microalgae samples were pyrolyzed on a TGA instrument under similar conditions (see Figure S3B). However, when temperature increased to 450°C (2nd fraction), the biochar yield decreased to 23% and the bio-oil increased to ~32%. From first and second fractions, a total 47% bio-oil was produced (sum of first and second fraction bio-oil yields since both are reported on a per gram ash-free biomass basis), which is close to the amount of bio-oil (~50%) achieved from single step microalgae pyrolysis at 450°C (see Figure 2A). Fractional pyrolysis with *ex situ* catalyst achieved a similar biochar yield as the one without catalyst, as expected. Moreover, 11 and 20% bio-oil was obtained from the first and second fractions, respectively. Overall, ~31% bio-oil was obtained from microalgae fractional pyrolysis in presence of *ex situ* catalyst. This total bio-oil is similar to the single step pyrolysis with *ex situ* catalyst (see Figure 2A). Thus, the fractional pyrolysis does not compromise net bio-oil yields and in presence of *ex situ* catalyst would provide a flexible downstream upgrading process, where each fraction can be upgraded separately.

#### Products Compositions

The bio-oil composition from each pyrolysis fraction is shown in Table 4 and the corresponding GC-MS chromatograms are shown in Figure S4 (catalyst-free) and Figure S5 (*ex situ* catalyst). Bio-oil from first fraction (320°C) of catalyst-free pyrolysis was mainly comprised of oxygenated compounds (e.g., acetic acid, ketone, aldehyde and furan), N-compounds (e.g., indole and pyrrole) and small amount of hydrocarbons. However, bio-oil from the second fraction contained mostly chemical compounds that were derived from triglyceride degradation/volatilization such as carboxylic acid (C_16_-C_18_ fatty acids), glyceride and hydrocarbons. This indicates majority of the lipid degradation products are collected in the second fraction.

**Table 4:** Bio-oil composition from fractional pyrolysis.

**Compound**	**Catalyst free**		**In presence of** ***ex-situ*** **catalyst**	
	**1st fraction**	**2nd fraction**	**1st fraction**	**2nd fraction**
Benzene	0.2	0.3	1.1	3.1
Toluene	–	0.3	2.2	5.9
Ethylbenzene	–	–	0.4	0.6
Xylene	–	0.1	1.2	3.9
C8+ aromatic	–	–	1.3	1.7
Naphthalene	–	–	0.9	2.4
Aliphatic hydrocarbons	0.7	3.7	2.7	0.9
Acetic acid	2.2	0.8		
Fatty acids	0.7	6.2	0.1	0.4
Other ketones/aldehydes	3.5	1.0	–	–
Furans	1.2	0.3	0.1	0.3
Indane	–	–	0.3	0.5
Indene	–	–	0.1	0.3
Other N-compounds	0.9	0.8	–	–
Alcohol	0.9	0.8	0.4	0.3
Fatty ester	0.4	1.8	–	–
Glycerides	0.7	12.0	–	–
Unidentified	3.3	3.43	0	0
Total liquid products	14.7	31.5	10.8	20.3
BTX^#^	0.2	0.8	4.5	12.9
Total aromatics	0.2	0.8	7.4	18.3
Aromatic carbon yield	n. a.	n. a.	10.7	26.5

# *Benzene, toluene and xylene*. *n. a., not applicable*. *The values are average of two experiments and are reported as weight percentage relative to dry, ash-free biomass*.

Bio-oil from fractional pyrolysis in presence of *ex situ* catalyst primarily contained C_6_-C_12_ aromatics, in particular benzene, toluene, xylene and naphthalene. The chemical compounds in first and second fractions were relatively similar, but the aromatic yields increased in the second fraction. Two contributing factors for the higher yields in the second step are possible. The first reason is the higher temperature of the second step which would increase catalyst activity. The second reason is the distinct reactants and reaction mechanisms in the two steps. In the first step HZSM-5 was likely converting oxygenates (such as aldehydes, ketones, furans, and alcohols) to aromatics whereas in the second step, the catalytic reactions were primarily with carbon-rich fatty acids and glycerides from lipid degradation/volatilization. In addition to the higher total aromatics in the second step, the BTX selectivity in the bio-oil from the second fraction was higher than the first fraction (see Figure S6). From first and second fraction, more than 17% BTX (relative to ash-free dry microalgae) was produced. A similar BTX yield was also achieved from the single-step pyrolysis with *ex situ* catalyst (Table 2). This demonstrates that integration of fractional pyrolysis with downstream *ex situ* catalytic upgrading allows carrying out the desired reaction chemistries on the vapor produced from each fraction.

Table 5 shows the ultimate analysis of the biochar from fractional pyrolysis of microalgae in presence of *ex situ* catalyst. As observed, the biochar from the first fraction has higher HHV than the second fraction due to higher C content. The biochar from the first fraction contained most of the C and N in the microalgae. C and N content after the second step remained similar to the one-step pyrolysis at 450°C (Table 3) showing that the fractional pyrolysis approach did not negatively impact the biochar composition, but allowed sperate recovery and upgrading of vapor fractions from energy dense lipids and O-/N- rich carbohydrates and starch.

**Table 5:** Elemental analyses of biochar obtained from fractional pyrolysis of microalgae in presence of *ex situ* catalyst.

**Ultimate analysis (wt. %)**	**1st fraction**		**2nd fraction**	
	**Dry**	**Dry, ash free**	**Dry**	**Dry, ash free**
C	48.2	70.7	31.0	56.6
H	4.3	6.2	1.1	2.1
N	3.1	4.5	2.4	4.3
C/N	18.4	18.4	15.3	15.3
H/C	1.1	1.1	0.4	0.4
HHV (MJ/kg)	19.1	31.2	16.4	20.8

## Conclusion

Microalgae pyrolysis experiments with *in situ* and *ex situ* HZSM-5 catalyst showed similarly high bio-oil yields suggesting that it was not necessary to intimately contact the catalyst with biomass. Rather, a close coupling of the pyrolysis vapors with the catalyst was sufficient to achieve high bio-oil yields and would allow easy recovery, regeneration and reuse of the catalyst as demonstrated in other studies with HZSM-5 (Cordero-Lanzac et al., 2018; Shirazi, 2018; Shirazi et al., 2019). A two-step fractional pyrolysis of microalgae integrated with *ex situ* catalyst was performed to upgrade the volatiles from microalgae's constituents separately. In absence of catalyst, the bio-oil from first fraction (pyrolyzed at 320°C) was mainly comprised of O-containing compounds from starch and protein degradation, while the bio-oil from the second fraction was rich in longer chain glycerides, fatty acids and aliphatics that were likely produced from degradation and/or volatilization of microalgae lipids. When the volatiles from each fraction were upgraded with an *ex situ* HZSM-5 catalyst, bio-oils from both fractions produced to aromatics (C_6_-C_12_) such as benzene, toluene, xylene and naphthalene with bio-oil yields and aromatics' selectivity similar to a single step catalytic pyrolysis at 450°C. These results indicate that when integrated with appropriate *ex situ* catalysts, oxygenates from starch degradation and higher calorific products from lipid degradation can be independently upgraded.

## Data Availability Statement

All datasets generated for this study are included in the article/Supplementary Material.

## Author Contributions

Experimental work and primary data analysis was performed by YS, SVi, was the primary supervisor of the research work. SVa co-supervised the research. All authors contributed to the article and approved the submitted version.

## Conflict of Interest

The authors declare that the research was conducted in the absence of any commercial or financial relationships that could be construed as a potential conflict of interest.

## References

[B1] Andrade L. A. Batista F. R. X. Lira T. S. Barrozo M. A. S. Vieira L. G. M. (2018). Characterization and product formation during the catalytic and non-catalytic pyrolysis of the green microalgae Chlamydomonas reinhardtii. Renewable Energy 119, 731–740. 10.1016/j.renene.2017.12.056

[B2] Azizi K. Moraveji M. K. Najafabadi H. A. (2018). A review on bio-fuel production from microalgal biomass by using pyrolysis method. Renewable Sustainable Energy Rev. 82, 3046–3059. 10.1016/j.rser.2017.10.033

[B3] Babu B. V. (2008). Biomass pyrolysis: a state-of-the-art review. Biofuels Bioproducts Biorefining 2, 393–414. 10.1002/bbb.92

[B4] Bridgwater A. V. (2012). Review of fast pyrolysis of biomass and product upgrading. Biomass Bioenergy 38, 68–94. 10.1016/j.biombioe.2011.01.048

[B5] Chaiwong K. Kiatsiriroat T. Vorayos N. Thararax C. (2013). Study of bio-oil and bio-char production from algae by slow pyrolysis. Biomass Bioenergy 56, 600–606. 10.1016/j.biombioe.2013.05.035

[B6] Chiaramonti D. Prussi M. Buffi M. Rizzo A. M. Pari L. (2017). Review and experimental study on pyrolysis and hydrothermal liquefaction of microalgae for biofuel production. Appl. Energy 185, 963–972. 10.1016/j.apenergy.2015.12.001

[B7] Cordero-Lanzac T. Ateka A. Pérez-Uriarte P. Castaño P. Aguayo A. T. Bilbao J. (2018). Insight into the deactivation and regeneration of HZSM-5 Zeolite catalysts in the conversion of Dimethyl ether to olefins. Ind. Eng. Chem. Res. 57, 13689–13702. 10.1021/acs.iecr.8b03308

[B8] Dong F. M. Hardy R. W. Haard N. F. Barrows F. T. Rasco B. A. Fairgrieve W. T. (1993). Chemical composition and protein digestibility of poultry by-product meals for salmonid diets. Aquaculture 116, 149–158. 10.1016/0044-8486(93)90005-J

[B9] Dong X. Chen Z. Xue S. Zhang J. Zhou J. Liu Y. (2013). Catalytic pyrolysis of microalga Chlorella pyrenoidosa for production of ethylene, propylene and butene. RSC Adv. 3, 25780–25787. 10.1039/C3RA43850C

[B10] Du Z. Ma X. Li Y. Chen P. Liu Y. Lin X.. (2013). Production of aromatic hydrocarbons by catalytic pyrolysis of microalgae with zeolites: catalyst screening in a pyroprobe. Bioresour. Technol. 139, 397–401. 10.1016/j.biortech.2013.04.053 23642438

[B11] Elkasabi Y. Chagas B. M. E. Mullen C. A. Boateng A. A. (2016). Hydrocarbons from spirulina pyrolysis bio-oil using one-step hydrotreating and aqueous extraction of HETEROATOM COmpounds. Energy Fuels 30, 4925–4932. 10.1021/acs.energyfuels.6b00473

[B12] Friedl A. Padouvas E. Rotter H. Varmuza K. (2005). Prediction of heating values of biomass fuel from elemental composition. Analytica Chimica Acta 544, 191–198. 10.1016/j.aca.2005.01.041

[B13] Furimsky E. (2013). Hydroprocessing challenges in biofuels production. Catalysis Today 217, 13–56. 10.1016/j.cattod.2012.11.008

[B14] Gautam R. Vinu R. (2018). Non-catalytic fast pyrolysis and catalytic fast pyrolysis of Nannochloropsis oculata using Co-Mo/gamma-Al_2_O_3_ catalyst for valuable chemicals. Algal Res. Biomass Biofuels Bioproducts 34, 12–24. 10.1016/j.algal.2018.06.024

[B15] Georgianna D. R. Mayfield S. P. (2012). Exploiting diversity and synthetic biology for the production of algal biofuels. Nature 488, 329–335. 10.1038/nature11479 22895338

[B16] Gong X. Zhang B. A. Zhang Y. Huang Y. F. Xu M. H. (2014). Investigation on pyrolysis of low lipid microalgae chlorella vulgaris and dunaliella salina. Energy Fuels 28, 95–103. 10.1021/ef401500z

[B17] Huber G. W. Corma A. (2007). Synergies between bio-and oil refineries for the production of fuels from biomass. Angew. Chemie Intern. Edn. 46, 7184–7201. 10.1002/anie.200604504 17610226

[B18] Huber G. W. Iborra S. Corma A. (2006). Synthesis of transportation fuels from biomass: chemistry, catalysts, and engineering. Chem. Rev. 106, 4044–4098. 10.1021/cr068360d 16967928

[B19] Jae J. Tompsett G. A. Foster A. J. Hammond K. D. Auerbach S. M. Lobo R. F. (2011). Investigation into the shape selectivity of zeolite catalysts for biomass conversion. J. Catal 279, 257–268. 10.1016/j.jcat.2011.01.019

[B20] Jarvis J. M. Sudasinghe N. M. Albrecht K. O. Schmidt A. J. Hallen R. T. Anderson D. B. (2016). Impact of iron porphyrin complexes when hydroprocessing algal HTL biocrude. Fuel 182, 411–418. 10.1016/j.fuel.2016.05.107

[B21] Jayanti S. Maheswaran K. Saravanan V. (2007). Assessment of the effect of high ash content in pulverized coal combustion. Appl. Math. Model 31, 934–953. 10.1016/j.apm.2006.03.022

[B22] Kubátová A. Št'ávová J. Seames W. S. Luo Y. Sadrameli S. M. Linnen M. J. (2011). Triacylglyceride thermal cracking: pathways to cyclic hydrocarbons. Energy Fuels 26, 672–685. 10.1021/ef200953d

[B23] Kumar G. Shobana S. Chen W.-H. Bach Q.-V. Kim S.-H. Atabani A. E. (2017). A review of thermochemical conversion of microalgal biomass for biofuels: chemistry and processes. Green Chem. 19, 44–67. 10.1039/C6GC01937D

[B24] Maddi B. (2014). Pyrolysis Strategies for Effective Utilization of Lignocellulosic and Algal Biomass. Toledo: University of Toledo.

[B25] Maddi B. Vadlamani A. Viamajala S. Varanasi S. (2017). Quantification of triglyceride content in oleaginous materials using thermo-gravimetry. J. Anal. Appl. Pyrolysis 128, 232–237. 10.1016/j.jaap.2017.10.006

[B26] Maddi B. Viamajala S. Varanasi S. (2011). Comparative study of pyrolysis of algal biomass from natural lake blooms with lignocellulosic biomass. Bioresour. Technol. 102, 11018–11026. 10.1016/j.biortech.2011.09.055 21983407

[B27] Maddi B. Viamajala S. Varanasi S. (2015a). Thermal Fractionation of Biomass of Non-Lignocellulosic Origin for Multiple High Quality Biofuels. United States patent application 61/413,177. Patent number US 8,927,240 B1 issued Jan 06, 2015.

[B28] Maddi B. Viamajala S. Varanasi S. (2015b). Thermal Fractionation of Biomass of Non-Lignocellulosic Origin for Multiple High Quality Biofuels, Continuation-in-Part. United States patent application 13/294,510. Patent number US 9,809,781 B1 issued Nov 07, 2017.

[B29] Maddi B. Viamajala S. Varanasi S. (2018). Pyrolytic fractionation: a promising thermochemical technique for processing oleaginous (Algal) biomass. ACS Sustainable Chem. Eng. 6, 237–247. 10.1021/acssuschemeng.7b02309

[B30] Miao X. Wu Q. Yang C. (2004). Fast pyrolysis of microalgae to produce renewable fuels. J. Anal. Appl. Pyrolysis 71, 855–863. 10.1016/j.jaap.2003.11.004

[B31] Mortensen P. M. Grunwaldt J. D. Jensen P. A. Knudsen K. G. Jensen A. D. (2011). A review of catalytic upgrading of bio-oil to engine fuels. Appl. Catalysis A 407, 1–19. 10.1016/j.apcata.2011.08.046

[B32] Mukarakate C. McBrayer J. D. Evans T. J. Budhi S. Robichaud D. J. Iisa K. (2015). Catalytic fast pyrolysis of biomass: the reactions of water and aromatic intermediates produces phenols. Green Chem. 17, 4217–4227. 10.1039/C5GC00805K

[B33] Mullen C. A. Tarves P. C. Boateng A. A. (2017). Role of potassium exchange in catalytic pyrolysis of biomass over ZSM-5: formation of alkyl phenols and furans. ACS Sustainable Chem. Eng. 5, 2154–2162. 10.1021/acssuschemeng.6b02262

[B34] Murugappan K. Mukarakate C. Budhi S. Shetty M. Nimlos M. R. Roman-Leshkov Y. (2016). Supported molybdenum oxides as effective catalysts for the catalytic fast pyrolysis of lignocellulosic biomass. Green Chem. 18, 5548–5557. 10.1039/C6GC01189F

[B35] Rezaei P. S. Shafaghat H. Daud W. M. A. W. (2014). Production of green aromatics and olefins by catalytic cracking of oxygenate compounds derived from biomass pyrolysis: a review. Appl. Catalysis A 469, 490–511. 10.1016/j.apcata.2013.09.036

[B36] Rizzo A. M. Prussi M. Bettucci L. Libelli I. M. Chiaramonti D. (2013). Characterization of microalga Chlorella as a fuel and its thermogravimetric behavior. Appl. Energy 102, 24–31. 10.1016/j.apenergy.2012.08.039

[B37] Sebestyén Z. Barta-Rajnai E. Czégény Z. Bhaskar T. Krishna B. B. May Z. (2016). Thermoanalytical characterization and catalytic conversion of deoiled micro algae and jatropha seed cake. Energy Fuels 30, 7982–7993. 10.1021/acs.energyfuels.6b01024

[B38] Shirazi Y. (2018). Production of Biofuels and Value-Added Chemicals from Oleaginous Biomass. Ph. D. DIssertation, University of Toledo.

[B39] Shirazi Y. Tafazolian H. Viamajala S. Varanasi S. Song Z. Heben M. J. (2017). High-yield production of fatty nitriles by one-step vapor-phase thermocatalysis of triglycerides. ACS Omega 2, 9013–9020. 10.1021/acsomega.7b01502 31457425 PMC6645552

[B40] Shirazi Y. Viamajala S. Varanasi S. (2016). High-yield production of fuel-and oleochemical-precursors from triacylglycerols in a novel continuous-flow pyrolysis reactor. Appl. Energy 179, 755–764. 10.1016/j.apenergy.2016.07.025

[B41] Shirazi Y. Viamajala S. Varanasi S. (2019). High-Yield Production of Fuels and Petro- and Oleo-Chemical Precursors from Vegetable Oils and Other Liquid Feedstocks in a Continuous-Flow Pyrolysis Reactor with or Without Catalysts. United States patent application 62 /377,958. Patent number US 10,190,058 B2 issued Jan 29, 2019.

[B42] Skorupskaite V. Makareviciene V. Gumbyte M. (2016). Opportunities for simultaneous oil extraction and transesterification during biodiesel fuel production from microalgae: a review. Fuel Processing Technol. 150, 78–87. 10.1016/j.fuproc.2016.05.002

[B43] Thangalazhy-Gopakumar S. Adhikari S. Chattanathan S. A. Gupta R. B. (2012). Catalytic pyrolysis of green algae for hydrocarbon production using H+ ZSM-5 catalyst. Bioresour. Technol. 118, 150–157. 10.1016/j.biortech.2012.05.080 22705518

[B44] Trinh T. N. Jensen P. A. Dam-Johansen K. Knudsen N. O. Sørensen H. R. Hvilsted S. (2013). Comparison of lignin, macroalgae, wood, and straw fast pyrolysis. Energy Fuels 27, 1399–1409. 10.1021/ef301927y

[B45] Urban B. Shirazi Y. Maddi B. Viamajala S. Varanasi S. (2017). Flash pyrolysis of oleaginous biomass in a fluidized-bed reactor. Energy Fuels 31, 8326–8334. 10.1021/acs.energyfuels.7b01306

[B46] Vadlamani A. (2016). Enhanced Biomass and Lipid Productivities of Outdoor Alkaliphilic Microalgae Cultures through Increased Media Alkalinity. Toledo: University of Toledo.

[B47] Vadlamani A. Viamajala S. Pendyala B. Varanasi S. (2017). Cultivation of microalgae at extreme alkaline pH conditions: a novel approach for biofuel production. ACS Sustainable Chem. Eng. 5, 7284–7294. 10.1021/acssuschemeng.7b01534

[B48] Venderbosch R. H. (2015). A critical view on catalytic pyrolysis of biomass. ChemSusChem. 8, 1306–1316. 10.1002/cssc.201500115 25872757

[B49] Verheijen F. Jeffery S. Bastos A. Van der Velde M. Diafas I. (2009). Biochar Application to Soils - A Critical Scientific Review of Effects on Soil Properties, Processes, and Functions. EUR 24099 EN. Luxembourg: Office for the Official Publications of the European Communities.

[B50] Wang K. Brown R. C. (2013). Catalytic pyrolysis of microalgae for production of aromatics and ammonia. Green Chem. 15, 675–681. 10.1039/C3GC00031A

[B51] Wang K. Brown R. C. Homsy S. Martinez L. Sidhu S. S. (2013). Fast pyrolysis of microalgae remnants in a fluidized bed reactor for bio-oil and biochar production. Bioresour. Technol. 127, 494–499. 10.1016/j.biortech.2012.08.016 23069615

[B52] Wang K. Johnston P. A. Brown R. C. (2014a). Comparison of in-situ and ex-situ catalytic pyrolysis in a micro-reactor system. Bioresour. Technol. 173, 124–131. 10.1016/j.biortech.2014.09.097 25299488

[B53] Wang K. Kim K. H. Brown R. C. (2014b). Catalytic pyrolysis of individual components of lignocellulosic biomass. Green Chem. 16, 727–735. 10.1039/C3GC41288A

[B54] Wang S. Dai G. Yang H. Luo Z. (2017). Lignocellulosic biomass pyrolysis mechanism: a state-of-the-art review. Progress Energy Combustion Sci. 62, 33–86. 10.1016/j.pecs.2017.05.004

[B55] Williams P. J. L. B Laurens L. M. L. (2010). Microalgae as biodiesel & biomass feedstocks: Review & analysis of the biochemistry, energetics & economics. Energy Environ. Sci. 3, 554–590. 10.1039/B924978H

[B56] Yu K. L. Lau B. F. Show P. L. Ong H. C. Ling T. C. Chen W.-H.. (2017). Recent developments on algal biochar production and characterization. Bioresour. Technol. 246, 2–11. 10.1016/j.biortech.2017.08.009 28844690

[B57] Yu X. Yang J. Lu H. Tu S.-T. Yan J. (2015). Energy-efficient extraction of fuel from Chlorella vulgaris by ionic liquid combined with CO_2_ capture. Appl. Energy 160, 648–655. 10.1016/j.apenergy.2015.04.074

