# Supplementary material for: In situ and Ex situ Catalytic Pyrolysis of Microalgae and Integration With Pyrolytic Fractionation
Source: Front Chem. 2020 Sep 10;8:786. doi: 10.3389/fchem.2020.00786 (PMC7533611; doi:10.3389/fchem.2020.00786)
Supplement: Supplementary file 1 [file Data_Sheet_1.PDF]

In situ and *ex situ* catalytic pyrolysis of microalgae and integration with pyrolytic fractionation

Yaser Shirazi[1], Sridhar Viamajala[1]* and Sasidhar Varanasi[1,2]

[1]*Department of Chemical and Environmental Engineering, University of Toledo, 2801 W. Bancroft St., Toledo OH 43606*
[2]*Department of Chemical Engineering, Manhattan College, 3825 Corlear Avenue -Leo 422*

*Corresponding Author:

Phone: 419-530-8094

Email: sridhar.viamajala@utoledo.edu

Table S1. Texture properties and acidity of HZSM-5 catalysts. Analytical methods are described in detail in our previous publication (Shirazi et al., 2017).

Properties →	Texture†			Acidity‡				
Catalyst ↓	Surface area¥ (m^2 g^{-1})	Pore size# (nm)	Pore volume@ (cm^3 g^{-1})	Total acidity (μmol g^{-1})%	1st acidity$ (μmol g^{-1})	2nd acidity* (μmol g^{-1})	$T_{max.1}$ (K)	$T_{max.2}$ (K)
HZSM-5	337.7	0.5	0.204	459.1	171.9	287.2	397.1	503

† Texture properties were measured by the ASAP2020 instrument.
‡ Acidity was measured by the NH_3-TPD method.
¥ BET surface area; # BJH Adsorption average pore diameter; @ Cumulative pore volume
% The values for acidity are in μmole of NH_3 desorption per gram of catalyst.
$ Measured from area of first peak; corresponds to medium/weak acid sites.
* Measured from area of second peak; corresponds to strong acid sites.
$T_{max.1}$: Temperature where maximum NH_3 desorption was observed in first peak from the NH_3 desorption versus temperature plot.
$T_{max.2}$: Temperature where maximum NH_3 desorption was observed in second peak from the NH_3 desorption versus temperature plot.

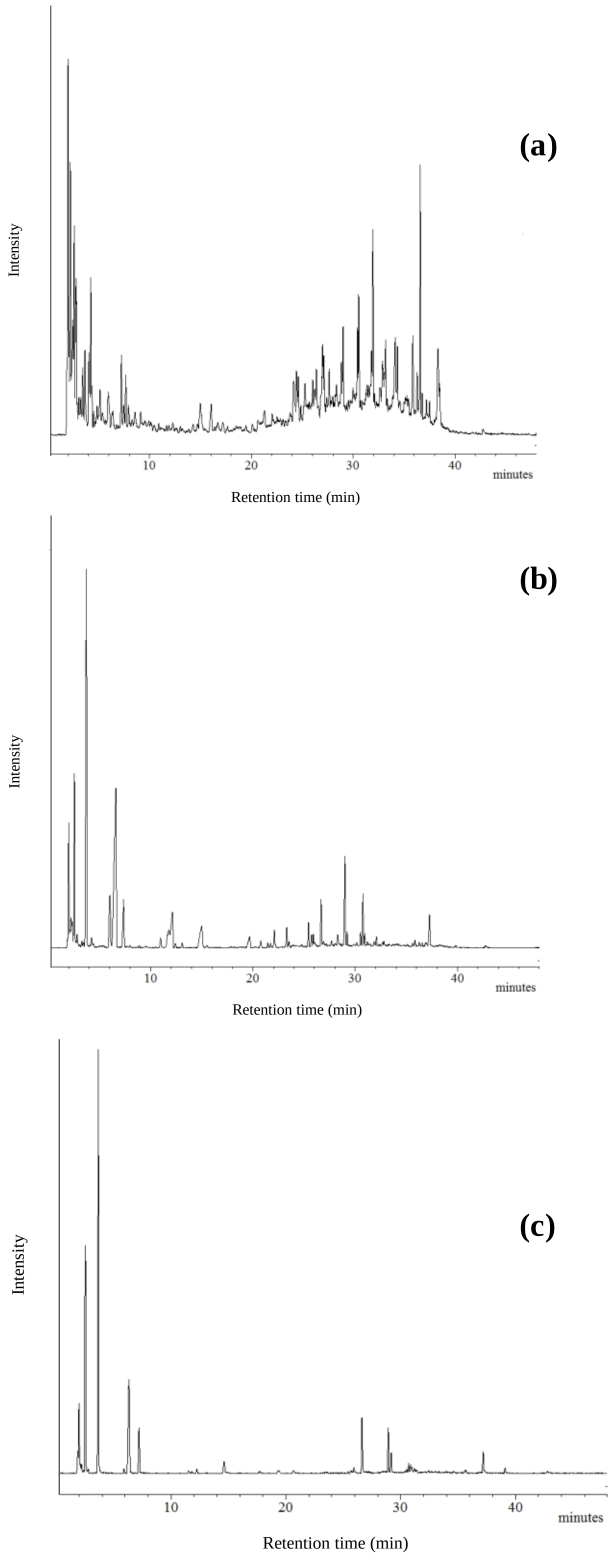

Figure S1. GC-MS chromatogram of bio-oil from pyrolysis in (a) absence of catalyst and in presence of (b) in-situ and (c) ex-situ catalyst.

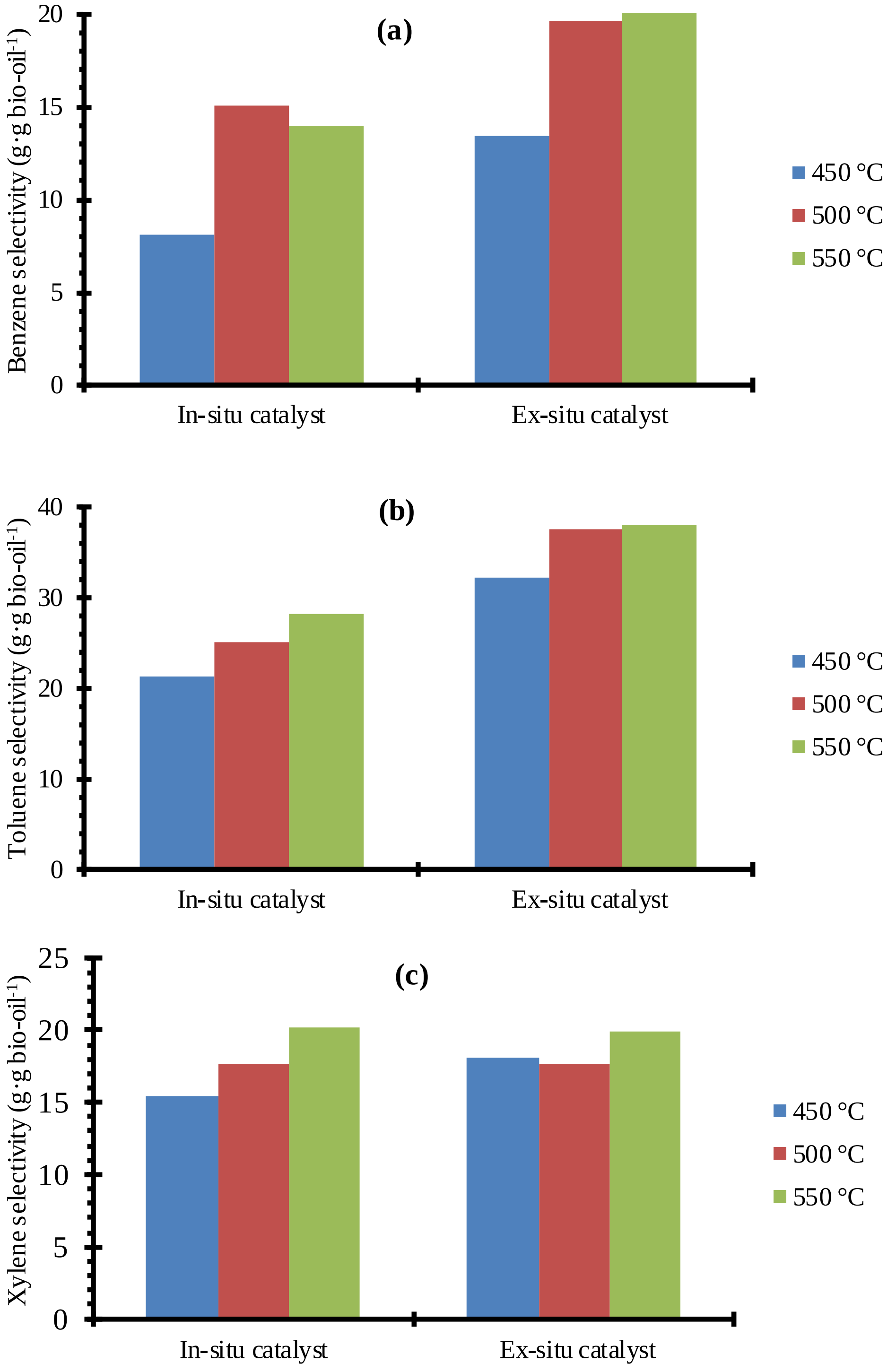

Figure S2. Selectivity of (a) benzene, (b) toluene and (c) xylene from *in situ* and *ex situ* catalytic pyrolysis of microalgae at tested temperatures.

(a)

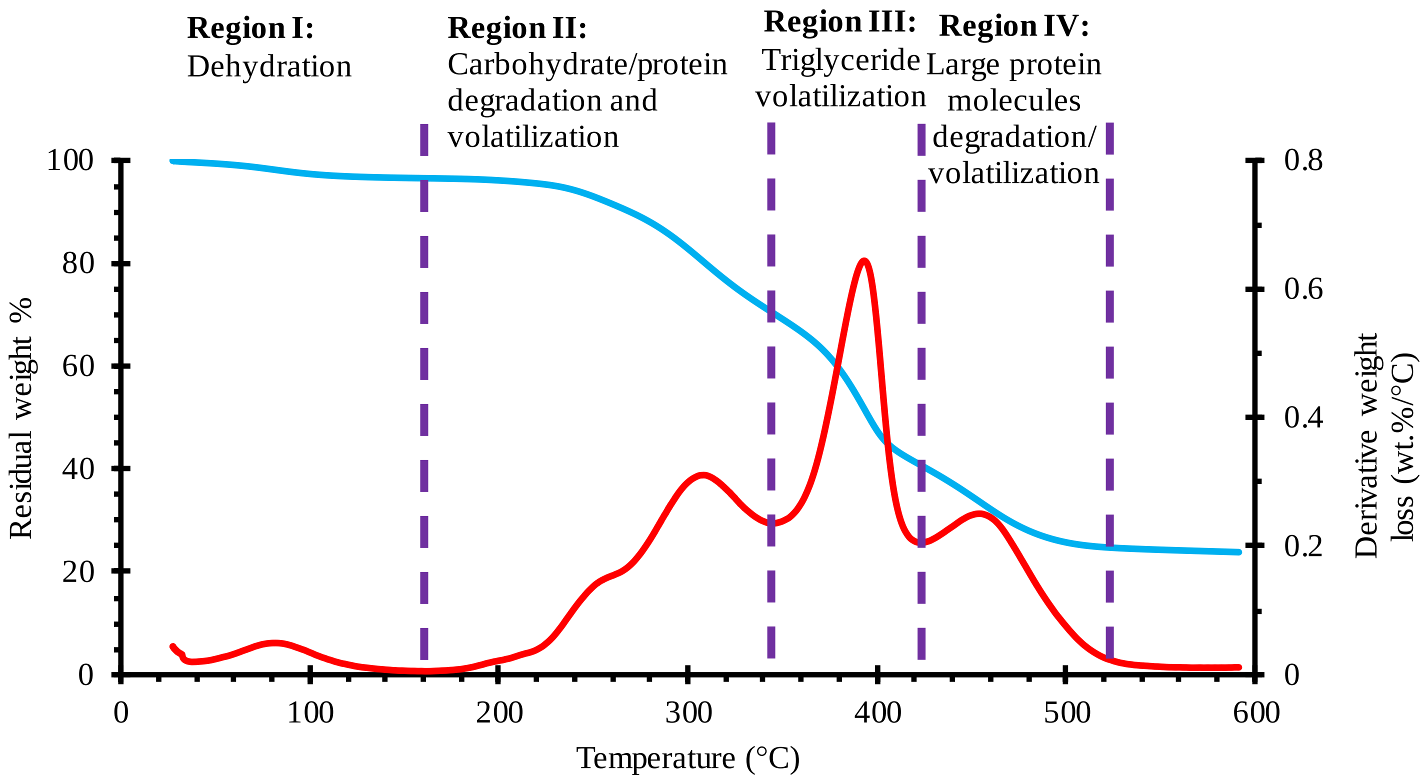

Region I:
Dehydration
Region II:
Carbohydrate/protein degradation and volatilization
Region III:
Triglyceride volatilization
Region IV:
Large protein molecules degradation/ volatilization
Residual weight %
Derivative weight loss (wt.%/°C)
Temperature (°C)
100
80
60
40
20
0
0.8
0.6
0.4
0.2
0
0
100
200
300
400
500
600

(b)

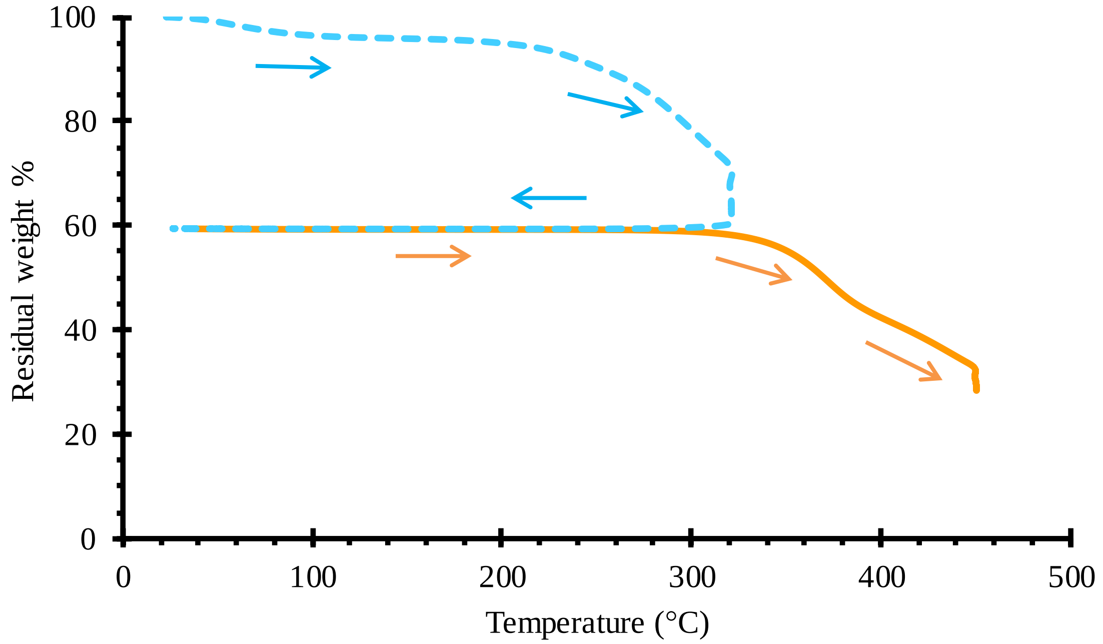

Residual weight %
Temperature (°C)
100
80
60
40
20
0
0
100
200
300
400
500

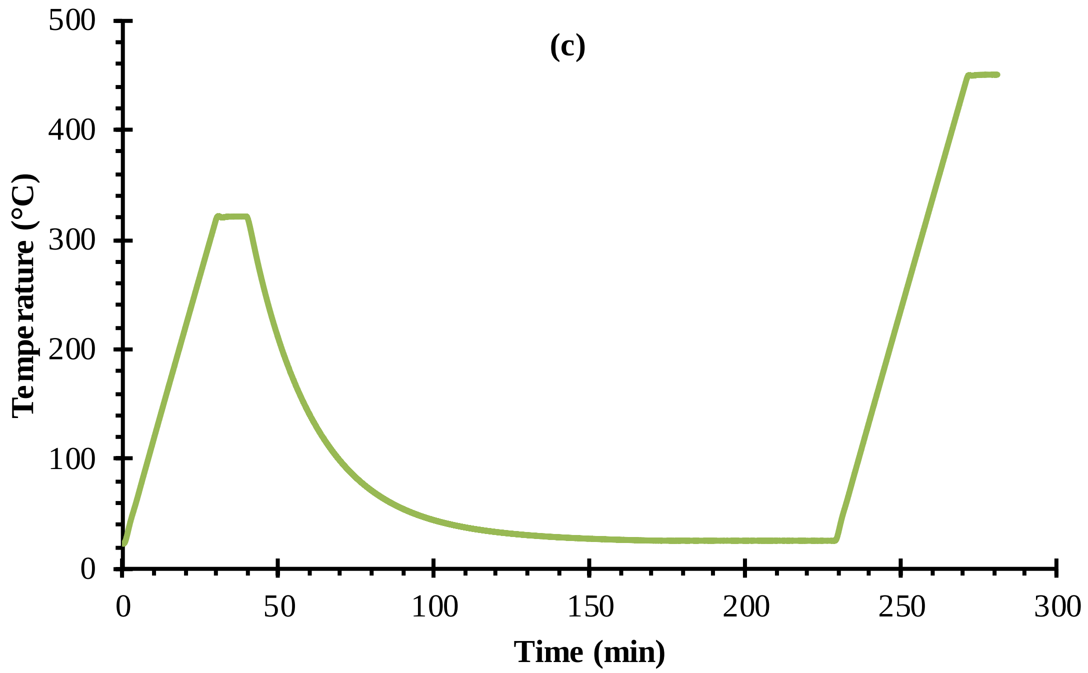

Figure S3. Thermogravimetric analysis of *Chlorella sorokiniana* SLA-04 biomass used in this study (a) thermogravimetric behavior of samples heated from room temperature to 600 °C at temperature ramp rate of 10 °C·min^{-1} and under N_2 atmosphere (b) fractional pyrolysis at 320 °C and 450 °C (c) temperature versus time profile in fractional pyrolysis.

Note 1: Figure S3b demonstrates a simulated fractional pyrolysis on TGA instrument. For this analysis, microalgae biomass was first heated under N_2 to 320 °C at 10 °C·min^{-1} temperature ramp and then held at 320 °C for 10 min. Then, the residue was cooled down to room temperature and reheated to 450 °C at 10 °C min^{-1} and held for 10 min.
Note 2: Figure S3c shows the temperature vs time profile during the simulated fractional pyrolysis experiments on the TGA.

Fractional pyrolysis simulation on TGA

Figure S3a shows the TGA analysis of microalgae, where it was heated from room temperature to 600 °C at 10 °C min^{-1} ramp and under N_2 atmosphere. Clearly, four regions of derivative weight loss can be observed from Figure S3a. *Region I* shows slight weight loss due to the dehydration of the water that likely trapped in microalgae cells and did not removed by freeze drying. *Region II* shows a weight loss from 160 to 340 °C contributed by decomposition of carbohydrate and protein (Maddi et al., 2017; Maddi et

al., 2018). The weight loss in *Region III* is due to triglyceride volatilization/degradation that occurs in temperature range of 345-430 °C (Maddi et al., 2017; Maddi et al., 2018). Interestingly, the derivative weight loss profile of microalgae showed another peak at temperature range of 430-530 °C (*Region IV*). Our previous TGA experiments of pure protein showed some protein (e.g. lysozyme) degradation occurs at higher temperature range (up to 500 °C) (Maddi et al., 2011). While we did not analyzed the protein type in microalgae in this study, the peak at *Region IV* can be due to degradation of larger protein molecules in microalgae. It is also possible that some of the bio-char (polycondensation products produced at the lower temperatures) decomposed at these high temperatures. Figure S3b demonstrates a simulated fractional pyrolysis on TGA – thermal degradation at 320 °C in the first step followed by a second step of pyrolysis at 450 °C. As observed in Figure S3b, the weight loss from the first fraction is nearly 40% and is likely due to the volatilization/decomposition of carbohydrate and protein constituents in microalgae. Interestingly, the residue from the first fraction did not show any weight loss when the temperature increased from room temperature up to 320 °C, which indicates that holding the microalgae at 320 °C for 10 min was sufficient to volatilize most of the carbohydrate and protein. Nearly 30% weight loss was achieved from the second fraction, which is close to the lipid content of the microalgae (Table 1). The TGA analysis shows that by fractional pyrolysis of microalgae first at 320 °C and then 450 °C, the products from carbohydrate and protein volatilization/decomposition can be recovered from the first fraction and the products from lipid constituents can be obtained from the second fraction.

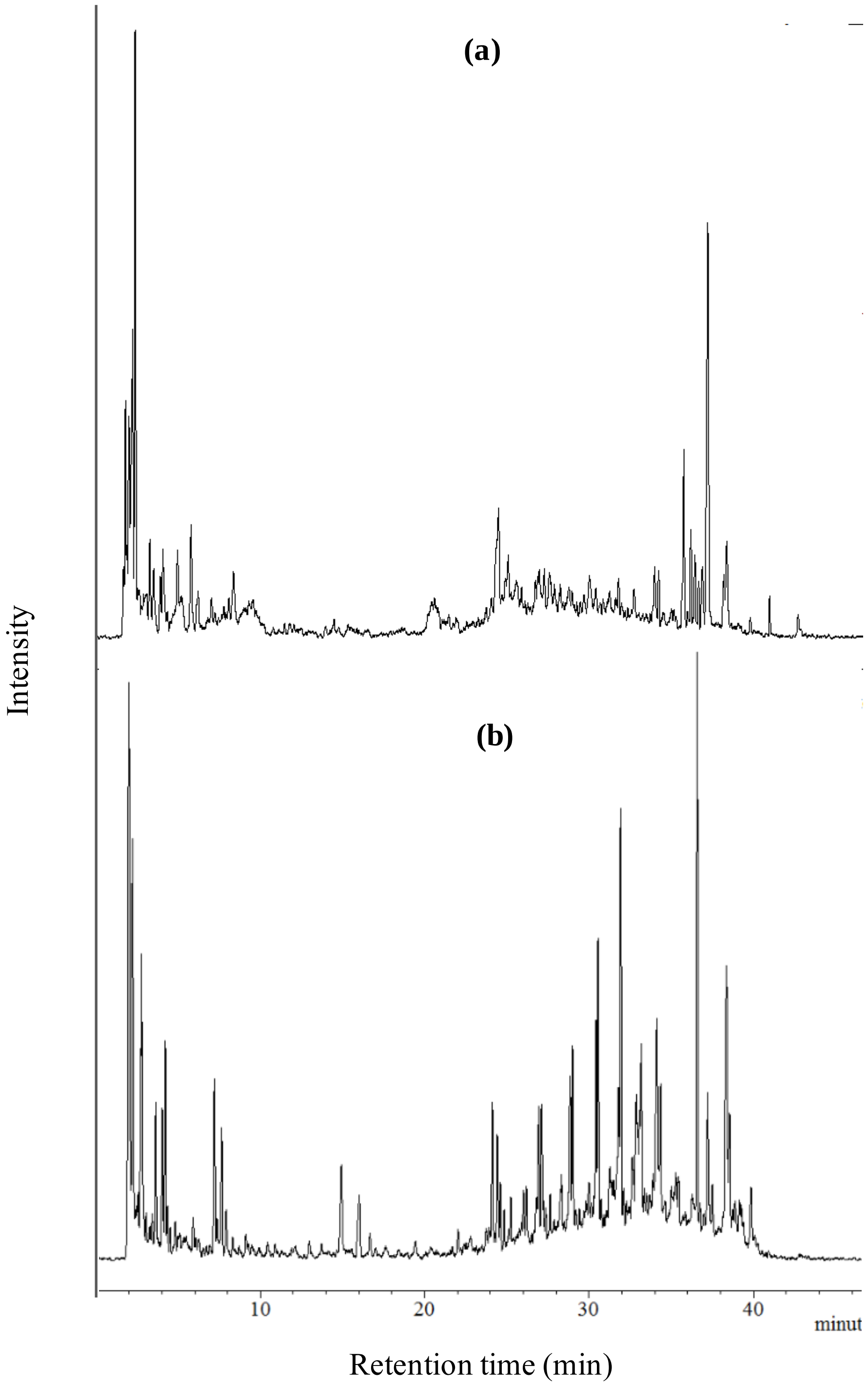

Figure S4. GC-MS chromatograms of bio-oils from (a) first (320 °C) and (b) second fractions (450 °C) of catalyst-free fractional pyrolysis.

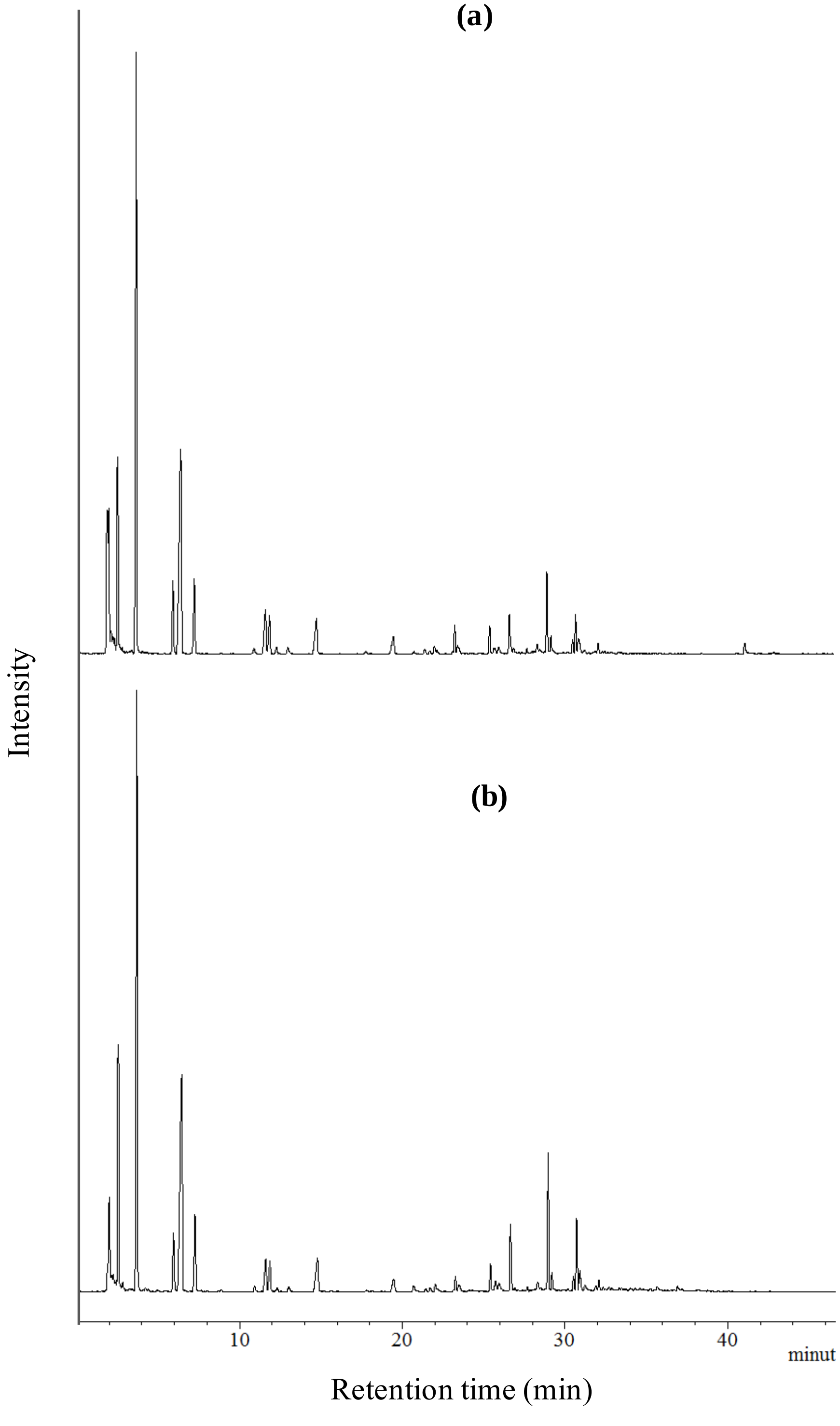

Figure S5. GC-MS chromatograms of bio-oils from (a) first (320 °C) and (b) second fractions (450 °C) of fractional pyrolysis in presence of *ex situ* catalyst.

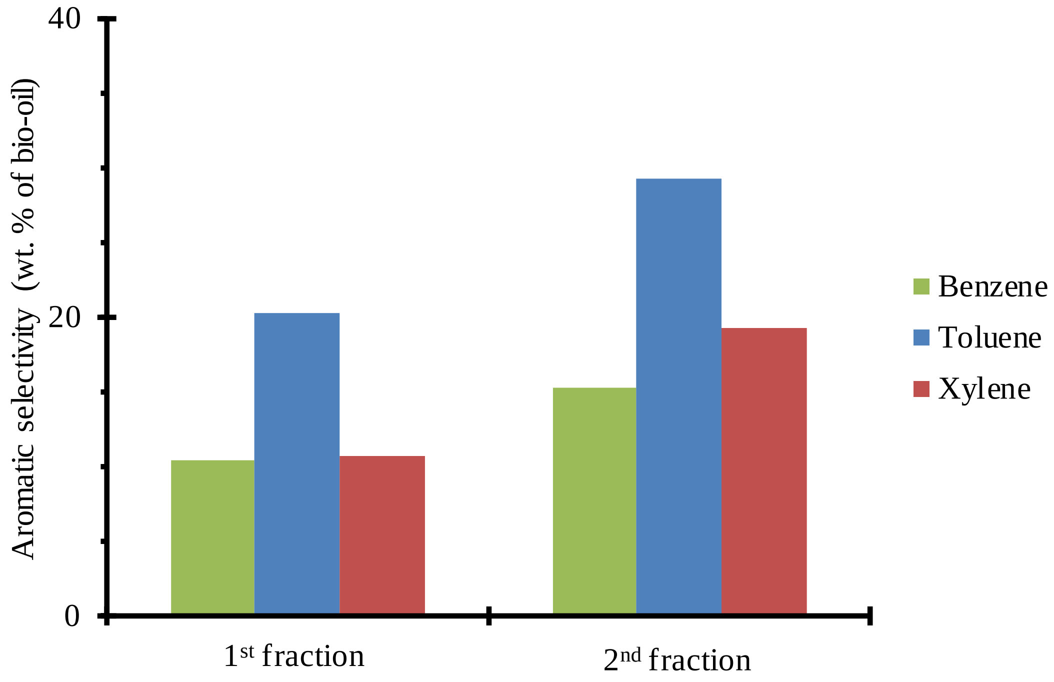

Figure S6. Selectivity of aromatics produced from microalgae fractional pyrolysis in presence of *ex situ* catalyst.

References

Maddi, B., Vadlamani, A., Viamajala, S., and Varanasi, S. (2017). Quantification of triglyceride content in oleaginous materials using thermo-gravimetry. *Journal of Analytical and Applied Pyrolysis* 128**,** 232-237. doi: https://doi.org/10.1016/j.jaap.2017.10.006.

Maddi, B., Viamajala, S., and Varanasi, S. (2011). Comparative study of pyrolysis of algal biomass from natural lake blooms with lignocellulosic biomass. *Bioresource Technology* 102(23)**,** 11018-11026. doi: http://dx.doi.org/10.1016/j.biortech.2011.09.055.

Maddi, B., Viamajala, S., and Varanasi, S. (2018). Pyrolytic Fractionation: A Promising Thermochemical Technique for Processing Oleaginous (Algal) Biomass. *ACS Sustainable Chemistry & Engineering* 6(1)**,** 237-247. doi: 10.1021/acssuschemeng.7b02309.

Shirazi, Y., Tafazolian, H., Viamajala, S., Varanasi, S., Song, Z., and Heben, M.J. (2017). High-Yield Production of Fatty Nitriles by One-Step Vapor-Phase Thermocatalysis of Triglycerides. *ACS Omega* 2(12)**,** 9013-9020. doi: 10.1021/acsomega.7b01502.
